# Supplementary material for: Trends in renal function in Northern Sweden 1986–2014: data from the seven cross-sectional surveys within the Northern Sweden MONICA study
Source: BMJ Open. 2023 Aug 30;13(8):e072664. doi: 10.1136/bmjopen-2023-072664 (PMC10471859; doi:10.1136/bmjopen-2023-072664)
Supplement: Supplementary data [file bmjopen-2023-072664supp001.pdf]

Trends in renal function in Northern Sweden 1986 – 2014: data from the seven cross-sectional surveys within the Northern Sweden MONICA study

Authors

Julia de Man Lapidoth[1], Johan Hultdin[2], P. Andreas Jonsson[1], Maria K Svensson[3], Maria Wennberg[4], Tanja Zeller[5,6], and Stefan Söderberg[1].

Affiliations

[1]Department of Public Health and Clinical Medicine, Medicine, Umeå University

[2]Department of Medical Biosciences, Clinical Chemistry, Umeå University

[3]Department of Medical Sciences, Renal Medicine, Uppsala University

[4]Department of Public Health and Clinical Medicine, Sustainable Health, Umeå University

[5]University Center of Cardiovascular Science, University Heart and Vascular Center Hamburg

[6]German Center of Cardiovascular Research (DZHK), Partner Seite Hamburg, Kiel, Lübeck, Hamburg/Germany

Supplementary tables 1-5

Supplementary table 1. Bivariate correlation analysis (Pearson)

Supplementary table 2. Bivariate correlation analysis (Pearson), survey 2004 excluded

Supplementary table 3. Univariable linear regression analysis

Supplementary table 4. Univariable linear regression analysis, survey 2004 excluded

Supplementary table 5. Multivariable linear regression analysis, survey 2004 excluded

de Man Lapidoth J, *et al. BMJ Open* 2024; 13:e072664. doi: 10.1136/bmjopen-2023-072664

Supplementary table 1. Bivariate correlation analysis (Pearson) between estimated glomerular filtration rate (eGFR) and clinical and biochemical variables including survey year

	$eGFR_{crea}$	$eGFR_{cysC}$
Survey year (1986–2014)	–0.142***	0
Age	–0.365***	–0.314***
BMI	–0.113***	–0.206***
Waist	–0.082***	–0.243***
Hip	–0.092***	–0.171***
SBP	–0.148***	–0.178***
DBP	–0.131***	–0.171***
Cholesterol	–0.126***	–0.146***
hsCRP	–0.206***	–0.324***
NTproBNP	–0.262***	–0.224***

$eGFR_{crea}$ and $eGFR_{cysC}$ according to the LM and CAPA formulas respectively and were Ln transformed. *** P <0.001. BMI=body mass index, SBP=systolic blood pressure, DBP=diastolic blood pressure, hsCRP= high sensitivity C–reactive protein, and NTproBNP=N–terminal fragment of the prohormone brain natriuretic peptide.

de Man Lapidoth J, *et al. BMJ Open* 2024; 13:e072664. doi: 10.1136/bmjopen-2023-072664

Supplementary table 2. Bivariate correlation analysis (Pearson), survey 2004 excluded

	$eGFR_{crea}$	$eGFR_{cysC}$
Survey year (1986–2014)	–0.222***	–0.094***
Age	–0.379***	–0.352***
BMI	–0.147***	–0.257***
Waist	–0.112***	–0.297***
Hip	–0.118***	–0.208***
SBP	–0.141***	–0.182***
DBP	–0.121***	–0.167***
Cholesterol	–0.115***	–0.139***
hsCRP	–0.199***	–0.327***
NTproBNP	–0.249***	–0.206***

$eGFR_{crea}$ and $eGFR_{cysC}$ according to the LM and CAPA formulas respectively and were Ln–transformed.

*** P <0.001. BMI=body mass index, SBP=systolic blood pressure, DBP=diastolic blood pressure, hsCRP=high sensitivity C–reactive protein, and NTproBNP=N–terminal fragment of the prohormone brain natriuretic peptide.

de Man Lapidoth J, *et al. BMJ Open* 2024; 13:e072664. doi: 10.1136/bmjopen-2023-072664

Supplementary table 3. Univariable linear regression analysis between two different estimated glomerular filtration rates (eGFR) and clinical and biochemical variables including survey year and county

	$eGFR_{crea}$		$eGFR_{cysC}$	
	**Unstandardized β**	**Standardized β**	**Unstandardized β**	**Standardized β**
Survey year (1986–2014)	–0.003***	–0.142***	0.000004	0
BMI (kg/m²)	–0.127***	–0.113***	–0.39***	–0.206***
Waist (cm)	–0.104***	–0.082***	–0.515***	–0.243***
Hip (cm)	–0.206***	–0.092***	–0.642***	–0.171***
SBP (mmHg)	–0.19***	–0.148***	–0.384***	–0.178***
DBP (mmHg)	–0.168***	–0.131***	–0.367***	–0.171***
Hypertension (Y/N)†	–0.04***	–0.125***	–0.076***	–0.141***
Diabetes mellitus (Y/N)‡	–0.088***	–0.08***	–0.112***	–0.061***
Active smoker (vs. never smoker)	0.024***	0.058***	–0.043***	–0.061***
Former smoker (vs. never smoker)	–0.013**	–0.033**	–0.023**	–0.035**
Cholesterol (mmol/L)	–0.105***	–0.126***	–0.204***	–0.146***
hsCRP (mg/L)	–0.032***	–0.206***	–0.085***	–0.324***
NTproBNP (ng/L)	–0.051***	–0.262***	–0.073***	–0.224***
Tertiary education (vs. primary school)	0.013**	0.030**	0.092***	0.130***
Secondary school (vs. primary school)	0.046***	0.120***	0.101***	0.159***
County (1=Västerbotten, 2=Norrbotten)	–0.012***	–0.033***	–0.016**	–0.027**

Unstandardized and standardized β–values are shown and $eGFR_{crea}$ and $eGFR_{cysC}$, respectively, are dependent variables in separate univariable analyses. $eGFR_{crea}$ and $eGFR_{cysC}$ according to the LM and CAPA formulas respectively and were Ln–transformed. * P <0.05, ** P < 0.01, *** P < 0.001. †Systolic blood pressure ≥140 mmHg and/or diastolic blood pressure ≥90 mmHg, and/or antihypertensive medication. ‡ Self–reported and/or use of glucose lowering medication. BMI=body mass index, SBP=systolic blood pressure, DBP=diastolic blood pressure, hsCRP=high sensitivity C–reactive protein, and NTproBNP=N–terminal fragment of the prohormone brain natriuretic peptide.

de Man Lapidoth J, *et al. BMJ Open* 2024; 13:e072664. doi: 10.1136/bmjopen-2023-072664

Supplementary table 4. Univariable linear regression analysis, survey 2004 excluded

	$eGFR_{crea}$		$eGFR_{cysC}$	
	**Unstandardized β**	**Standardized β**	**Unstandardized β**	**Standardized β**
Survey year (1986–2014)	–0.004***	–0.222***	–0.003***	–0.094***
Age (years)	–0.006***	–0.379***	–0.008***	–0.352***
Sex (1=male, 2=female)	–0.014***	–0.041***	0.084***	0.153***
BMI (kg/m²)	–0.164***	–0.147***	–0.45***	–0.257***
Waist (cm)	–0.139***	–0.112***	–0.579***	–0.297***
Hip (cm)	–0.259***	–0.118***	–0.715***	–0.208***
SBP (mmHg)	–0.178***	–0.141***	–0.36***	–0.182***
DBP (mmHg)	–0.153***	–0.121***	–0.331***	–0.167***
Hypertension (Y/N)†	–0.043***	–0.131***	–0.079***	–0.155***
Diabetes mellitus (Y/N)‡	–0.103***	–0.095***	–0.123***	–0.072***
Active smoker (vs. never smoker)	0.031***	0.075***	–0.036***	–0.057***
Former smoker (vs. never smoker)	–0.010*	–0.027*	–0.020**	–0.033**
Cholesterol (mmol/L)	–0.094***	–0.115***	–0.178***	–0.139***
hsCRP (mg/L)	–0.03***	–0.199***	–0.078***	–0.327***
NTproBNP (ng/L)	–0.047***	–0.249***	–0.061***	–0.206***
Tertiary education (vs. primary school)	0.000	0.001	0.067***	0.102***
Secondary school (vs. primary school)	0.033***	0.089***	0.082***	0.140***
County (1=Västerbotten, 2=Norrbotten)	–0.02***	–0.058***	–0.024***	–0.044***

$eGFR_{crea}$ and $eGFR_{cysC}$ according to the LM and CAPA formulas, respectively and were Ln–transformed. Unstandardized and standardized [illegible]–values are shown and $eGFR_{crea}$ and $eGFR_{cysC}$, respectively, are dependent variables in univariable analyses. * P <0.05, ** P < 0.01, *** P < 0.001. †Systolic blood pressure ≥140 mmHg and/or diastolic blood pressure ≥90 mmHg, and/or antihypertensive medication. ‡ Self–reported and/or use of glucose lowering medication. BMI=body mass index, SBP=systolic blood pressure, DBP=diastolic blood pressure, hsCRP=high sensitivity C–reactive protein, and NTproBNP=N–terminal fragment of the prohormone brain natriuretic peptide.

Supplementary table 5. Multivariable linear regression analysis, survey 2004 excluded

	$eGFR_{crea}$		$eGFR_{cysC}$	
	**Unstandardized β**	**Standardized β**	**Unstandardized β**	**Standardized β**
Survey	–0.005***	–0.26***	–0.004***	–0.128***
BMI (kg/m²)	–0.009	–0.008	–0.197***	–0.112***
Hypertension (Y/N)†	–0.015***	–0.045***	–0.021***	–0.041***
Diabetes mellitus (Y/N)‡	–0.065***	–0.06***	–0.036*	–0.021*
Active smoker (vs. never smoker)	0.025***	0.05***	–0.028***	–0.043***
Former smoker (vs. never smoker)	–0.003	–0.008	0.000	0.000
Cholesterol (mmol/L)	–0.124***	–0.152***	–0.133***	–0.103***
hsCRP (mg/L)	–0.019***	–0.127***	–0.054***	–0.227***
NT–pro–BNP (ng/L)	–0.038***	–0.201***	–0.045***	–0.152***
Tertiary education (vs. primary school)	0.006	0.015	0.041***	0.062***
Secondary school (vs. primary school)	0.032***	0.085***	0.054***	0.091***
County (1=Västerbotten, 2=Norrbotten)	–0.013***	–0.038***	–0.004	–0.007

$eGFR_{crea}$ and $eGFR_{cysC}$ according to the LM and CAPA formulas respectively and were Ln–transformed. Unstandardized and standardized ⍰–values are shown, and $eGFR_{crea}$ and $eGFR_{cysC}$, respectively, are dependent variables in separate multivariable analysis. * P <0.05, ** P < 0.01, *** P < 0.001. †Systolic blood pressure ≥140 mmHg and/or diastolic blood pressure ≥90 mmHg, and/or antihypertensive medication. ‡ Self–reported and/or use of glucose lowering medication. BMI=body mass index, SBP=systolic blood pressure, DBP=diastolic blood pressure, hsCRP=high sensitivity C–reactive protein, and NTproBNP=N–terminal fragment of the prohormone brain natriuretic peptide.
