# Supplementary material for: Trends in renal function in Northern Sweden 1986–2014: data from the seven cross-sectional surveys within the Northern Sweden MONICA study
Source: BMJ Open. 2023 Aug 30;13(8):e072664. doi: 10.1136/bmjopen-2023-072664 (PMC10471859; doi:10.1136/bmjopen-2023-072664)
Supplement: Author's manuscript [file bmjopen-2023-072664.draft_revisions.pdf]

BMJ Open

BMJ Open is committed to open peer review. As part of this commitment we make the peer review history of every article we publish publicly available.

When an article is published we post the peer reviewers' comments and the authors' responses online. We also post the versions of the paper that were used during peer review. These are the versions that the peer review comments apply to.

The versions of the paper that follow are the versions that were submitted during the peer review process. They are not the versions of record or the final published versions. They should not be cited or distributed as the published version of this manuscript.

BMJ Open is an open access journal and the full, final, typeset and author-corrected version of record of the manuscript is available on our site with no access controls, subscription charges or pay-per-view fees (http://bmjopen.bmj.com).

If you have any questions on BMJ Open's open peer review process please email info.bmjopen@bmj.com

BMJ Open

Deterioration in renal function in Northern Sweden between 1986 and 2014 – A population–based study

Journal:	*BMJ Open*
Manuscript ID	bmjopen-2023-072664
Article Type:	Original research
Date Submitted by the Author:	11-Feb-2023
Complete List of Authors:	de Man Lapidoth , Julia; Umeå Universitet Medicinska fakulteten, Dept of Public Health and Clinical Medicine<br>Hultdin, Johan; Umeå University, Department of Medical Biosciences, Clinical Chemistry<br>Jonsson, P. Andreas; Umeå Universitet Medicinska fakulteten, Dept of Public Health and Clinical Medicine<br>Eriksson Svensson, Maria; Uppsala University, Renal medicine<br>Wennberg, Maria; Umeå University, Department of Public Health and Clinical Medicine<br>Zeller, Tanja ; University of Hamburg, Clinic for general and interventional Cardiology<br>Söderberg, Stefan ; Umeå University, Department of Public Health and Clinical Medicine
Keywords:	EPIDEMIOLOGY, PUBLIC HEALTH, Chronic renal failure < NEPHROLOGY

SCHOLARONE™
Manuscripts

I, the Submitting Author has the right to grant and does grant on behalf of all authors of the Work (as defined in the below author licence), an exclusive licence and/or a non-exclusive licence for contributions from authors who are: i) UK Crown employees; ii) where BMJ has agreed a CC-BY licence shall apply, and/or iii) in accordance with the terms applicable for US Federal Government officers or employees acting as part of their official duties; on a worldwide, perpetual, irrevocable, royalty-free basis to BMJ Publishing Group Ltd ("BMJ") its licensees and where the relevant Journal is co-owned by BMJ to the co-owners of the Journal, to publish the Work in this journal and any other BMJ products and to exploit all rights, as set out in our licence.

The Submitting Author accepts and understands that any supply made under these terms is made by BMJ to the Submitting Author unless you are acting as an employee on behalf of your employer or a postgraduate student of an affiliated institution which is paying any applicable article publishing charge ("APC") for Open Access articles. Where the Submitting Author wishes to make the Work available on an Open Access basis (and intends to pay the relevant APC), the terms of reuse of such Open Access shall be governed by a Creative Commons licence – details of these licences and which Creative Commons licence will apply to this Work are set out in our licence referred to above.

Other than as permitted in any relevant BMJ Author's Self Archiving Policies, I confirm this Work has not been accepted for publication elsewhere, is not being considered for publication elsewhere and does not duplicate material already published. I confirm all authors consent to publication of this Work and authorise the granting of this licence.

For peer review only

Deterioration in renal function in Northern Sweden between 1986 and 2014 – A population–based study

Short title: Deterioration in renal function over time

Authors list

Julia de Man Lapidoth[1], Johan Hultdin[2], P. Andreas Jonsson[1], Maria K Svensson[3], Maria Wennberg[4], Tanja Zeller[5], and Stefan Söderberg[1].

Affiliations

1 Department of Public Health and Clinical Medicine, Medicine, Umeå University

2 Department of Medical Biosciences, Clinical Chemistry, Umeå University

3 Department of Medical Sciences, Renal Medicine, Uppsala University

4 Department of Public Health and Clinical Medicine, Sustainable Health, Umeå University

5 University Center of Cardiovascular Science, University Heart and Vascular Center Hamburg

Correspondence: Stefan Söderberg, Department of Public Health and Clinical Medicine, Medicine, Umeå University. Phone: +46 70 319 38 03, email: stefan.soderberg@umu.se

Word count and supplementary material

Main manuscript – 3787 (except references and tables), abstract – 233, references – 45, and tables – 3. 5 tables and STROBE attached as supplementary material

For peer review only

Abstract:

Objective: The prevalence of chronic kidney disease (CKD) is increasing globally and CKD and cardiovascular disease (CVD) are closely linked. CKD and CVD share several risk factors (RF), such as diabetes, hypertension, obesity, and smoking, and the prevalence of these RF has changed during the last decades, and we aimed to study the effect on renal function over time.

Design: Repeated cross-sectional studies

Setting: The two northernmost counties (Norr- and Västerbotten) in Sweden.

Participants: Within the MONitoring Trends and Determinants of CArdiovascular Disease (MONICA) study, seven surveys were performed between 1986 and 2014, including participants aged 25 to 64 years (n=10,185).

Interventions: None

Measures: Information on anthropometry, blood pressure and cardiovascular risk factors was collected. Creatinine and cystatin C were analyzed in stored blood samples and the estimated glomerular filtration rate (eGFR) calculated using the creatinine-based Lund–Malmö (LM) revised and Chronic Kidney Disease Epidemiology Collaboration (CKD–EPI) ($eGFR_{crea}$) equations, as well as the cystatin C-based Caucasian, Asian, Paediatric and Adult cohort (CAPA) equation ($eGFR_{cysC}$). Renal function over time was analyzed using uni– and multivariable linear regression models.

Results: Renal function, both $eGFR_{crea}$ and $eGFR_{cysC,}$ decreased over time (both $P < 0.001$), and differed between counties and sexes. In a multivariable analysis, study year remained

For peer review only - http://bmjopen.bmj.com/site/about/guidelines.xhtml

For peer review only

inversely associated with both $eGFR_{crea}$ and $eGFR_{cysC}$ (both $P<0.001$) even after adjustment for classical cardiovascular RF.

Conclusion: Renal function seems to have deteriorated in Northern Sweden between 1986 – 2014.

Key words: estimated glomerular filtration rate; renal function; creatinine; cystatin C; cardiovascular risk factors

For peer review only

Strengths and limitations of this study

- Study design, selection of participants and measurements consistent over time
- Population based study
- Creatinine and Cystatin C measured in the same laboratory during the same period
- Several risk factors were self-reported
- Possible residual confounding

For peer review only

Introduction

The prevalence of chronic kidney disease (CKD) is increasing globally, and deaths attributed to CKD constituted 4.6% of all deaths in 2017.[1] The SCREAM cohort represents, to date, the largest patient cohort to estimate the burden and implications of CKD in Sweden, and an estimated 6% of the adult Stockholm population accessing healthcare has CKD defined as an eGFR < 60 ml/min/1.73m^2.[2]

Decreased renal function is associated with an increased risk for cardiovascular events, including cardiovascular death.[3, 4] This is partly due to shared risk factors for CKD and CVD, for example diabetes, hypertension, obesity, and smoking.[5-7] Other possible mechanisms such as systemic inflammation, renal anemia, and endothelial cell dysfunction may contribute.[8] In addition, data suggest that subjects with CVD and CKD get less secondary prevention after a cardiovascular event.[9, 10] Medical treatment for patients with end–stage kidney disease (ESKD) constitutes 7% of the total cost of Medicare in the US, though these patients only comprised less than 1% of the total Medicare population. At the same time, patients with CKD and ESKD receive over 20 % of the total Medicare fee–for–service cost (including treating comorbidities and other healthcare services that may be unrelated).[11]

In Northern Sweden, the prevalence of hypertension, smoking, and hypercholesterolemia have decreased, whereas obesity has increased.[12] In parallel, the use of antihypertensive drugs has increased.[12] Furthermore, low socioeconomic status is associated with decreased renal function, possibly due to the clustering of other cardiovascular risk factors.[13] Longitudinally, there are thus several factors that might have impacted renal function both negatively and positively over time, and the potential net effect is not known.

For peer review only - http://bmjopen.bmj.com/site/about/guidelines.xhtml

This study aimed to investigate the development on renal function over 28 years in Northern Sweden, considering the concomitant changes in the prevalence of major cardiovascular risk factors and other factors that may impact renal function over time.

Methods

This study is based on seven cross-sectional studies performed within the framework of the Northern Sweden MONICA (multinational MONItoring of trends and determinants in CArdiovascular disease) study.[14] The World Health Organizations' MONICA project was initiated in the mid–eighties, and the first survey in the two most northern counties of Sweden (Västerbotten and Norrbotten) was performed in 1986. Altogether seven population–based surveys using similar methods have been conducted (in 1986, 1990, 1994, 1999, 2004, 2009, and 2014). All surveys were performed during the same months (January – April) to eliminate seasonal variations. The participants have been randomly selected to represent the population and make the different surveys comparable. Two examination teams collected all data using standardized methods to the extent it is possible. Participants were invited if aged between 25–64 years in 1986 and 1990 (n=2,000 per survey), and 25–74 years from 1994 (n=2,500 per survey). Altogether 11,924 subjects participated (mean participation rate 72 %, range 81–61%), with a declining rate for each survey. The majority of participants were Swedish Caucasians, with a small minority of Finnish and Sami heritage.

Patient and public involvement

No patient involvement

Questionnaires

For peer review only - http://bmjopen.bmj.com/site/about/guidelines.xhtml

All participants answered questions about diabetes mellitus (yes/no/don't know). Those who answered yes or had diabetes medications were considered as having diabetes. Questions regarding smoking involve active smoking, sporadic smoking, former smoking, former sporadic smoking, and non–smoker. The answers were recoded into active smoking (active and sporadic smokers), former smoking (former and former sporadic smokers), and non–smoker. Medications taken regularly were grouped according to ATC–code. All participants with angiotensin–converting enzyme inhibitor (ACEi), angiotensin receptor blocker (ARB) or mineralocorticoid receptor antagonist (MRA) treatment were identified.

The level of education was registered and divided into three groups: tertiary education (university), secondary school, and primary school.

Measurements

Height was measured without shoes using a calibrated scale with a measuring rod attached to the scale or mounted on the wall. The participants wore thin clothing to measure their weight, and a calibrated scale was used (a balance scale 1986–1994, from 1999, an electrical scale was used). The weight was estimated to nearest 0,2 kilograms. Body mass index was calculated as weight divided by height squared.[15] The waist and hip circumference were measured with a tape measure without clothes between the iliac crest and the lowest rib, over the symphysis bone, and over the part of the hips with the greatest width. With this information, the waist–hip ratio was calculated.[15] After five minutes of rest in the sitting position, blood pressure was measured in the right arm twice at a three-minute interval, and the mean value was calculated. The Hawksley random–zero sphygmomanometer was used between 1986 and 2004, and from 2009 the Omron M7–device was used.[16] Hypertension

For peer review only - http://bmjopen.bmj.com/site/about/guidelines.xhtml

was defined as systolic blood pressure ≥140 mmHg, diastolic blood pressure ≥ 90 mmHg, or the use of antihypertension medication for at least two weeks before the survey.

Blood samples

Blood samples were drawn after fasting for at least 4 hours. Total cholesterol levels were determined at a regional laboratory within 24 hours using an enzymatic method in 1986–1994. From 1999, a dry–chemistry method was used.[17]

In 2015, stored serum samples from all surveys (stored frozen at –80° C) were sent to Hamburg/Germany, as part of the BiomarCaREproject, for further analysis of Cystatin C, Creatinine, N–terminal fragment of the prohormone brain natriuretic peptide (NTproBNP), and high-sensitivity C-reactive protein (hsCRP).[18] For approximately 50% of the samples from 1990, EDTA plasma was used instead of serum.

Cystatin C was analyzed using a latex immunoturbidimetric assay and creatinine with a Jaffe / kinetic assay (Kinetic Alkaline Picrate), performed on an Architect C8000 system (Abbott, Abbot Park, IL, USA). Measurement ranges were for creatinine 17.7–3271 µmol/L with a LOQ of 4.4 µmol/L. Creatinine was IDMS (isotope dilution mass spectrometry) traceable (NIST SRM 967). Cystatin C had a measurement range of 0.05 – 9.6 mg/L, and the limit of quantification (LOQ) was 0.05 mg/L. hsCRP was analyzed with a highly sensitive latex immunoturbidimetric assay, CRP16vario, and NTproBNP was measured using Electrochemiluminescence sandwich immunoassay ECLIA on an ELECSYS 2010 platform (Roche Diagnostics, Basel, Switzerland).

In addition, in 2009 and 2014 creatinine was measured locally with an IDMS traceable method.

For peer review only - http://bmjopen.bmj.com/site/about/guidelines.xhtml

Statistical analyses

Two different formulas for calculating the creatinine–based eGFR ($eGFR_{crea}$) were used, the Lund–Malmö (LM) revised formula[19] and the Chronic Kidney Disease Epidemiology Collaboration (CKD–EPI) formula from 2009.[20] To estimate cystatin C–based eGFR ($eGFR_{cysC}$), the Caucasian, Asian, Paediatric, and Adult cohort (CAPA) formula[21] was used. For the CKD–EPI calculation, all participants were labeled as Caucasians.

- LM revised formula: $e^{X - 0.0158 \times Age + 0.438 \times \ln(Age)}$, if female and creatinine <150 X = 2.50 + 0.0121 × (150–creatinine), if female and creatinine ≥150 X = 2.50 – 0.926 × Ln(creatinine/150), if male and creatinine < 180 X = 2.56 + 0.00926 × (180 – creatinine), if male and creatinine ≥ 180 X = 2.56 – 0.926 × Ln(creatinine/180)
- CKD–EPI formula from 2009: $141 \times \min\left(\frac{Scr}{\kappa,1}\right)^{\alpha} \times \max\left(\frac{Scr}{\kappa,1}\right)^{-1.209} \times 0.993^{Age}$ $\times 1.018$ [if female] $\times 1.159$ [if black], Scr = serum creatinine, κ = 0.7 (male) and 0.9 (female), α = –0.411 (male) and –0.329 (female), min = the minimum of Scr/κ or 1 and max = the maximum of Scr/κ or 1.
- CAPA = $130 \times (cystatin\ C^{-1.069}) \times (age^{-0.117}) - 7$

Using the Dubois–Dubois formula,[22] absolute eGFR was also calculated.

$$\frac{eGFR \times (0.007184 \times length^{0.725} \times weight^{0.425}}{1.73}$$

Since inclusion criteria changed in 1994, participants aged 65 years or more were excluded to make surveys comparable. Descriptive data included mean values with 95% confidence intervals and medians with interquartile ranges. If necessary, some variables were ln–transformed to improve normality, and geometric means were calculated. Pearson's

For peer review only - http://bmjopen.bmj.com/site/about/guidelines.xhtml

correlation analyses were performed. To evaluate associations between eGFR and year of survey adjusted for major cardiovascular risk factors, uni– and multivariable linear regression analyses were performed.

Plasma concentrations of creatinine and Cystatin C were markedly higher in 2004. Therefore, both results including and excluding data from 2004 are presented.

Most continuous variables were ln–transformed to improve normality. Number of missing data are reported in Table 1 and no imputation was done.

To test the robustness of the results, hsCRP and NTproBNP and data from 2004 were excluded from the multivariable model in separate steps, and different formulas for calculation of eGFR were used.

Age and sex were not included in the linear regression or correlation analyses, as these variables are included in the equations of eGFR. For categorical variables with more than two groups, dummy variables were created.

The SPSS statistical program version 28 was used (IBM Corporation, New York, NY, USA).

For peer review only

Results

Baseline data from 7 surveys are presented in Supplementary Table 1. The study included 10,185 participants with a mean age (95% confidence interval) of 46 (24–65) years, and 51% were women.

The prevalence of major cardiovascular risk factors changed between 1986 and 2014: total cholesterol, systolic blood pressure, and active smoking decreased, whereas BMI increased.

hsCRP, NTproBNP, and the proportion of the participants using drugs interacting with the RAAS system (ACEi, ARB, and MRA) increased as well (0 to 10.2 %). A dramatic increase in the proportion of participants with higher education, an important socioeconomic factor, was seen over 28 years.

We found that $eGFR_{crea}$ (both revised LM and the CKD–EPI) and $eGFR_{cysC}$ (CAPA) decreased over time (Supplementary Table 2 and Figure 1). Overall, a more pronounced decrease in renal function (eGFR) was observed over time in Norrbotten county compared to Västerbotten county. Women had higher $eGFR_{cysC}$, but a lower $eGFR_{crea}$ compared to men. The $eGFR_{crea}$ measured locally was lower in 2014 compared to 2009, 84.0 (83.3 – 84.7) versus 84.8 (84.2 – 85.4), P=0.03.

In the correlation analysis, study year associated inversely with $eGFR_{crea}$ (Table 1, *P*=<0.001), and with both $eGFR_{crea}$ and $eGFR_{cysC}$ if data from 2004 were excluded (Supplementary Table 3, *P*=<0.001). Age, anthropometric measures, blood pressure, total cholesterol, and CRP and NTproBNP were inversely associated with both $eGFR_{crea}$ and $eGFR_{cysC}$ irrespectively if data from 2004 were included or not.

The univariable linear regression analyses displayed the same pattern (Table 2 and Supplementary Table 4). Study year associated inversely with $eGFR_{crea}$(*P*=<0.001) and with both $eGFR_{crea}$ and $eGFR_{cysC}$ if data from 2004 were excluded (*P*=<0.001). Furthermore, female sex associated with lower $eGFR_{crea}$ (*P*=0.001) but with higher $eGFR_{cysC}$ (*P*=<0.001). Higher attained educational level associated with higher $eGFR_{crea}$ and higher $eGFR_{cysC}$. Active smokers had higher $eGFR_{crea}$ but lower $eGFR_{cysC}$. These results based on the LM and CAPA formulas were almost identical if the CKD–EPI formula was used instead or if absolute eGFR was calculated (data not shown).

For peer review only - http://bmjopen.bmj.com/site/about/guidelines.xhtml

In the final multivariable linear regression models, study year remained inversely associated with both $eGFR_{crea}$ and $eGFR_{cysC}$ (Table 5, both *P*=<0.001). This was seen irrespective of including or excluding hsCRP and NTproBNP (data not shown) or data from 2004 (Supplementary Table 5).

Lower $eGFR_{crea}$ associated with diabetes, hypertension, and higher cholesterol, and with higher CRP and NTproBNP levels, and lower $eGFR_{cysC}$ associated with increasing BMI, hypertension, higher cholesterol, active smoking, and with higher CRP and NTproBNP levels. In contrast, active smoking associated with higher $eGFR_{crea}$. A higher educational level remained positively associated with both $eGFR_{crea}$ and $eGFR_{cysC}$, whereas county did not associate with eGFR after adjustments. Using the CKD–EPI equation gave almost identical results (data not shown).

Study year remained inversely associated with $eGFR_{crea}$ and $eGFR_{cysC}$ also if hypertension was replaced with systolic and diastolic blood pressure, if the use of ACEi, ARB, and MRA was added, or if the waist and hip circumferences were added (data not shown). High blood pressure and the use of RAAS blocking agents (ACEi, ARB, and MRA) were all independently associated with lower $eGFR_{crea}$ and $eGFR_{cysC}$ (data not shown).

For peer review only

Discussion

In this study we found that renal function assessed as $eGFR_{crea}$ and $eGFR_{cysC}$ seem to have decreased over 28 years in Northern Sweden. This was also seen after adjustment for concomitant changes in the prevalence of major cardiovascular risk factors and other known cardiovascular risk factors that may impact renal function over time. Notably, the renal function differed between the studied counties, possibly due to varying cardiovascular risk

For peer review only - http://bmjopen.bmj.com/site/about/guidelines.xhtml

profiles, as the difference did not remain after adjustments. Possibly, the ongoing primary–care intervention program in Västerbotten may have had an impact.[23] We also found that women have higher $eGFR_{cysC}$ but lower $eGFR_{crea}$ than men, which is not in line with earlier studies showing that male gender is usually associated with a lower $eGFR_{crea.}$[24]

To our knowledge, a similar study of renal function over a long time in a study with well–defined population–based samples has not been done before. Previous studies have focused on the changes in renal function in the same cohort over time, mainly showing deteriorating renal function as a result of increasing age.[24]

We adjusted for several factors to try to explain the deteriorating renal function. However, the decline in eGFR remained an independent finding in the multivariable analysis. This finding may be worrying as reduced renal function is associated with an increased mortality risk.[25] Hypertension and smoking contribute to a faster decline in renal function,[6] and the use of antihypertensive medications has increased in northern Sweden,[26] including RAAS–inhibitors ACEi, ARB, and MRA. These medications are known to initially decrease the eGFR due to a reduction of glomerular hyperfiltration[27] and could partly explain an observed decrease in renal function over time. The observed inverse relationship between the use of RAAS–inhibitors could also be due to co–linearity with the conditions *(i.e*. hypertension and renal protection) that they are used for. However and importantly, the long–term treatment with these medications preserve renal function.[28]

According to a previous study on this population, the reported energy intake from protein increased slightly from 14 to 16 E% from 1990 until 2014. It is unlikely that this small increase had any large impact on the finding of increasing creatinine.[29] In addition,

For peer review only - http://bmjopen.bmj.com/site/about/guidelines.xhtml

increasing creatinine and a lower estimated eGFR is further supported by the finding of decreasing renal function when using cystatin C to estimate renal function.

Another potential explanation could be increased exposure to environmental pollutants,[30] but previous studies have shown a decrease in lead in the population and a stable amount of cadmium.[31] However, numerous other pollutants that could potentially affect renal function, which are not analyzed in this study.

Northern Sweden also has a high incidence and prevalence of the viral infection nephropathia epidemica (Puumala virus),[32] which is a hemorrhagic fever with a renal syndrome (HFRS). The long–term effects are still unknown, and earlier studies have shown contradictory results if the infection causes irreversible long-term kidney damage.[33, 34]

Smoking affected $eGFR_{crea}$ and $eGFR_{cysC}$ differentially, which has been presented earlier.[35] It has been suggested that smoking causes glomerular hyperfiltration[36] affecting creatinine more but also that sarcopenia may contribute to lower creatinine values in smokers. Possibly, smoking per se may increase cystatin C levels[37] which is supported by studies showing a decrease in cystatin C following smoking cessation.[38]

This study shows that higher education is associated with better renal function, which is in line with other previous studies.[39] Lower socioeconomic status (SES) is associated with a higher incidence of CKD,[40] and a higher risk of chronic renal failure.[41] Several indicators for SES have been used, such as educational attainment, household, or individual income, or geocoordinates. However, similar results related to renal function have been shown irrespective of how SES has been defined.[39]

The strength of this study is that renal function is measured using two different endogenous markers, creatinine and cystatin C, which are affected by different confounding factors[42, 43]

For peer review only - http://bmjopen.bmj.com/site/about/guidelines.xhtml

and both endogenous markers show the same trend indicating that renal function seems to decline over time. An additional strength is that data is collected from a randomly selected, large cohort, representing the general population at the time of the survey, which makes the results applicable to the general population. One additional strength is that all creatinine and cystatin C analyses for the different years were performed within the same period, at the same laboratory, using the same method.

There are several limitations as well. Some of the blood samples have been frozen for a long period, the earliest from 1986, which could affect the results of the biochemical analyses. Prolonged storage time could result in the degradation of biomarkers and sublimation of water from the frozen samples altering concentrations of the biomarkers. Still, the finding of a reduction of both $eGFR_{crea}$ and $eGFR_{cysC}$ over time suggests that there is an actual reduction in renal function (eGFR). Furthermore, it should be emphasized that the samples have been stored in –80° degrees from sampling to analysis without thawing. The method to analyze creatinine, the Jaffe/kinetic assay, is not specific and other compounds (for example, hemoglobin F, certain proteins, glucose, and ketone bodies) produce Jaffe–like chromogens (also known as pseudocreatinines or non–creatinine chromogens). To adjust for this potential overestimation, compensated analyses can be used. Since all blood samples are analyzed using the same method, it is possible to use this method to investigate renal function over time in the population. However, we saw variations in levels of biomarkers related to survey year, the 2004 results in particular. We, therefore, repeated all analyses after excluding 2004 data which gave similar results. Quality indicates have been scrutinized, but no obvious reason has been found for this discrepancy.

For peer review only - http://bmjopen.bmj.com/site/about/guidelines.xhtml

Other limitations lay in that some of the data included in this study are self–reported (diabetes, smoking, medication), which makes recall bias impossible to eliminate. The participation rate has been very good in the MONICA surveys (72%), which is high compared to other population–based surveys internationally. There is always a selection in this kind of studies, and healthier and older individuals are more likely to participate. Since eGFR is an estimation of renal function and not a direct measurement, it is possible that these results are not exclusively based on a decreasing renal function but could also be affected by changes in living conditions and measurements. One known risk factor is alcohol intake.[44] This is not included in this study due to the lack of reliability of self–reported alcohol intake but a previous study on dietary trends in this population found an increase in reported energy from alcohol over time.[29] We also lack information about medications bought over the counter (OTC) such as non–steroidal anti–inflammatory drug (NSAID) that may impact renal function both short term and long-term.[45] Finally, the results are based on a predominately Caucasian population in Northern Scandinavia, and the generalizability to other ethnicities and contexts is unknown.

Conclusion

In this study we found that estimated renal function seems to have deteriorated in Northern Sweden independently of changes in cardiovascular risk factors and socioeconomic status.

For peer review only

Acknowledgement

The authors thank the MONICA project, scientific leaders, and participants through the years, as well as the Department of Biobank Research at Umeå University (https://www.umu.se/en/biobank–research–unit/) for providing data and blood samples.

The authors also want to thank the MORGAM/BiomarCaRe team in Hamburg and Helsinki for the extensive analyses of biomarkers. A special thanks to Professor Kari Kuulasma in Helsinki, Finland and to Dr Stefan Blankenberg in Hamburg, Germany.

For peer review only

Figure legend

Renal function in Northern Sweden between 1986 and 2014 expressed as eGFR (geometric mean) based on the Lund–Malmö (LM) revised formula and the Chronic Kidney Disease Epidemiology Collaboration (CKD–EPI) formula based on creatinine, and the Caucasian, Asian, Paediatric, and Adult cohort (CAPA) based on Cystatin C.

For peer review only

For peer review only - http://bmjopen.bmj.com/site/about/guidelines.xhtml

Contributorship statement

SS is principal investigator for the Northern Sweden MONICA study and is responsible for the repeated population-based surveys and the collection of data and samples. SS initiated this study and drafted the main hypothesis. TZ is responsible for the analyses of Creatinine and Cystatin C and related qualitative checks. JDL made the primary analyses and drafted the manuscript, which has been carefully interpreted and revised by all authors. All authors have read and approved the final version of the manuscript. SS and JDL have full access to the dataset.

Competing interests' statement

MKS reports no conflict of interest or competing interests to declare related to this project. Provided expertise in ad boards and given lectures for Amgen, AstraZeneca, Boehringer Ingelheim and GSK. Been a clinical trialist and scientific collaborations with Bayer, MSD, Novo Nordisk, AstraZeneca and Vifor Pharma. SS reports speaker honoraria from Actelion Ltd. All others declare no disclosures.

Funding

The Northern Sweden MONICA study was funded by the county councils in Norr– and Västerbotten, and by Umeå university. The BiomarCaRE Project was funded by the European Union Seventh Framework Programme (FP7/2007–2013) under grant agreement No. HEALTH–F2–2011–278913. Dr Lapidoth was supported by ALF from the county council of Västerbotten.

Data sharing statement

Data from the Northern MONICA surveys are not freely available according to GDPR due to the presence of individual data. However, pseudonymized data can be shared after ethical

For peer review only - http://bmjopen.bmj.com/site/about/guidelines.xhtml

evaluation and formal request (https://www.umu.se/en/biobankresearch–unit/research/access–to–samples–anddata/access–to–nsdd/).

Ethical approval statement

All participants have signed informed consent for future research using collected data and blood samples. The Ethics Review Board in Umeå has approved the study protocol according to the Declaration of Helsinki (for surveys 2013/97–31 and for biochemical analyses 2012–280–32M).

For peer review only

For peer review only - http://bmjopen.bmj.com/site/about/guidelines.xhtml

References

1. Purcell CA, Abebe M, Adebayo OM, et al. Global, regional, and national burden of chronic kidney disease, 1990–2017: a systematic analysis for the Global Burden of Disease Study 2017. *The Lancet (British edition)*. 2020;395:709-33.
2. Gasparini A, Evans M, Coresh J, et al. Prevalence and recognition of chronic kidney disease in Stockholm healthcare. *Nephrology, dialysis, transplantation*. 2016;31:2086-94.
3. Rangaswami J, Bhalla V, Blair JEA, et al. Cardiorenal Syndrome: Classification, Pathophysiology, Diagnosis, and Treatment Strategies: A Scientific Statement From the American Heart Association. *Circulation (New York, NY)*. 2019;139:e840-e78.
4. Go AS, Chertow GM, Fan D, et al. Chronic kidney disease and the risks of death, cardiovascular events, and hospitalization. *N Engl J Med*. 2004;351:1296-305.
5. Tuttle KR, Alicic RZ, Duru OK, et al. Clinical Characteristics of and Risk Factors for Chronic Kidney Disease Among Adults and Children: An Analysis of the CURE-CKD Registry. *JAMA network open*. 2019;2:e1918169.
6. Toyama T, Kitagawa K, Oshima M, et al. Age differences in the relationships between risk factors and loss of kidney function: a general population cohort study. *BMC nephrology*. 2020;21:477-.
7. Pe De J, Jc V, Sj P-S, et al. Obesity and target organ damage: the kidney. *International Journal of Obesity*. 2002;26:S21.
8. Gansevoort RTD, Correa-Rotter RP, Hemmelgarn BRP, et al. Chronic kidney disease and cardiovascular risk: epidemiology, mechanisms, and prevention. *The Lancet (British edition)*. 2013;382:339-52.

For peer review only

9. Fox CS, Muntner P, Chen AY, et al. Use of Evidence-Based Therapies in Short-Term Outcomes of ST-Segment Elevation Myocardial Infarction and Non-ST-Segment Elevation Myocardial Infarction in Patients With Chronic Kidney Disease: A Report From the National Cardiovascular Data Acute Coronary Treatment and Intervention Outcomes Network Registry. *Circulation (New York, NY)*. 2010;121:357-65.

10. Szummer K, Lundman P, Jacobson SH, et al. Relation between renal function, presentation, use of therapies and in-hospital complications in acute coronary syndrome: data from the SWEDEHEART register. *Journal of internal medicine*. 2010;268:40-9.

11. United States Renal Data System. 2022 USRDS Annual Data Report: Epidemiology of kidney disease in the United States.

12. Eriksson M, Holmgren L, Janlert U, et al. Large improvements in major cardiovascular risk factors in the population of northern Sweden: the MONICA study 1986-2009. *J Intern Med*. 2011;269:219-31.

13. Weldegiorgis M, Smith M, Herrington WG, et al. Socioeconomic disadvantage and the risk of advanced chronic kidney disease: Results from a cohort study with 1.4 million participants. *Nephrology, dialysis, transplantation*. 2020;35:1562-70.

14. Stegmayr B, Lundberg V and Asplund K. The events registration and survey procedures in the Northern Sweden MONICA Project. *Scandinavian Journal of Public Health*. 2003;31:9-17.

15. Lilja M, Eliasson M, Stegmayr B, et al. Trends in Obesity and Its Distribution: Data From the Northern Sweden MONICA Survey, 1986–2004. *Obesity (Silver Spring, Md)*. 2008;16:1120-8.

For peer review only

For peer review only - http://bmjopen.bmj.com/site/about/guidelines.xhtml

16. Eriksson M, Carlberg B and Jansson J-H. Comparison of blood pressure measurements between an automated oscillometric device and a Hawksley random-zero sphygmomanometer in the northern Sweden MONICA study. *Blood pressure monitoring*. 2012;17:164-70.

17. Eliasson M, Janlert U, Jansson JH, et al. Time trends in population cholesterol levels 1986–2004: influence of lipid-lowering drugs, obesity, smoking and educational level. The northern Sweden MONICA study. *Journal of internal medicine*. 2006;260:551-9.

18. Zeller T, Hughes M, Tuovinen T, et al. BiomarCaRE: rationale and design of the European BiomarCaRE project including 300,000 participants from 13 European countries. *European journal of epidemiology*. 2014;29:777-90.

19. Nyman U, Grubb A, Larsson A, et al. The revised Lund-Malmö GFR estimating equation outperforms MDRD and CKD-EPI across GFR, age and BMI intervals in a large Swedish population. *Clinical Chemistry and Laboratory Medicine (CCLM)*. 2014;52:815-24.

20. Levey AS, Stevens LA, Schmid CH, et al. A new equation to estimate glomerular filtration rate. *Annals of internal medicine*. 2009;150:604-12.

21. Grubb A, Horio M, Hansson L-O, et al. Generation of a New Cystatin C–Based Estimating Equation for Glomerular Filtration Rate by Use of 7 Assays Standardized to the International Calibrator. *Clinical chemistry (Baltimore, Md)*. 2014;60:974-86.

22. Du Bois D and Du Bois EF. A formula to estimate the approximate surface area if height and weight be known. *Archives of internal medicine (1960)*. 1916;17:863-71.

23. Eliasson M, Eriksson M, Lundqvist R, et al. Comparison of trends in cardiovascular risk factors between two regions with and without a community and primary care prevention programme. *European journal of preventive cardiology*. 2018;25:1765-72.

For peer review only

24. Eriksen BO and Ingebretsen OC. The progression of chronic kidney disease: A 10-year population-based study of the effects of gender and age. *Kidney International*. 2006;69:375.

25. Turin TC, Coresh J, Tonelli M, et al. Change in the estimated glomerular filtration rate over time and risk of all-cause mortality. *Kidney international*. 2013;83:684-91.

26. Torma E, Carlberg B, Eriksson M, et al. Long term trends in control of hypertension in the Northern Sweden MONICA study 1986-2009. *BMC Public Health*. 2015;15.

27. Cortinovis M, Perico N, Ruggenenti P, et al. Glomerular hyperfiltration. *Nature reviews Nephrology*. 2022;18:435-51.

28. Zhang F, Liu H, Liu D, et al. Effects of RAAS Inhibitors in Patients with Kidney Disease. *Current hypertension reports*. 2017;19:72-.

29. Törmä J, Lundqvist R, Eliasson M, et al. Comparison of dietary trends between two counties with and without a cardiovascular prevention programme: a population-based cross-sectional study in northern Sweden. *Public health nutrition*. 2022;25:1835-43.

30. Copur S, Ucku D and Kanbay M. Increase in the global burden of chronic kidney disease: might it be attributable to air pollution? *Clinical kidney journal*. 2022;15:1800-2.

31. Wennberg M, Lundh T, Sommar JN, et al. Time trends and exposure determinants of lead and cadmium in the adult population of northern Sweden 1990–2014. *Environmental research*. 2017;159:111-7.

32. Bo N and James WL. Epidemiology of Nephropathia Epidemica in Sweden. *The Journal of infectious diseases*. 1987;155:269-76.

33. Latus J, Schwab M, Tacconelli E, et al. Clinical course and long-term outcome of hantavirus-associated nephropathia epidemica, Germany. *Emerging infectious diseases*. 2015;21:76-83.

For peer review only

34. Mäkelä S, Ala-Houhala I, Mustonen J, et al. Renal function and blood pressure five years after Puumala virus-induced nephropathy. *Kidney international*. 2000;58:1711-8.

35. Eric LK, Jacobien CV, Donna S, et al. Factors influencing serum cystatin C levels other than renal function and the impact on renal function measurement. *Kidney International*. 2004;65:1416.

36. Mickelsson M, Söderström E, Stefansson K, et al. Smoking tobacco is associated with renal hyperfiltration. *Scandinavian journal of clinical and laboratory investigation*. 2021;81:622-8.

37. Yamada Y, Noborisaka Y, Ishizaki M, et al. Different association of cigarette smoking with GFR estimated from serum creatinine and that from serum cystatin C in the general population. *Clinical and experimental nephrology*. 2015;19:669-77.

38. Funamoto M, Shimizu K, Sunagawa Y, et al. Serum Cystatin C, a Sensitive Marker of Renal Function and Cardiovascular Disease, Decreases After Smoking Cessation. *Circulation reports*. 2019;1:623-7.

39. Vart PMPH, Gansevoort RTMDP, Joosten MMP, et al. Socioeconomic Disparities in Chronic Kidney Disease. *American journal of preventive medicine*. 2015;48:580-92.

40. Park S, Lee S, Kim Y, et al. Causal effects of education on chronic kidney disease: a Mendelian randomization study. *Clinical Kidney Journal*. 2021;14:1932-8.

41. Fored CM, Ejerblad E, Fryzek JP, et al. Socio-economic status and chronic renal failure: a population-based case-control study in Sweden. *Nephrology, dialysis, transplantation*. 2003;18:82-8.

42. Baxmann AC, Ahmed MS, Marques NC, et al. Influence of Muscle Mass and Physical Activity on Serum and Urinary Creatinine and Serum Cystatin C. *Clinical journal of the American Society of Nephrology*. 2008;3:348-54.

For peer review only

43. Stevens LA, Schmid CH, Greene T, et al. Factors other than glomerular filtration rate affect serum cystatin C levels. *Kidney international*. 2009;75:652-60.

44. Epstein M. Alcohol's impact on kidney function. *Alcohol research & health*. 1997;21:84.

45. Hörl WH. Nonsteroidal anti-inflammatory drugs and the kidney. *Pharmaceuticals*. 2010;3:2291-321.

For peer review only

For peer review only - http://bmjopen.bmj.com/site/about/guidelines.xhtml

Table 1. Bivariate correlation analysis (Pearson) between estimated glomerular filtration rate (eGFR) and clinical and biochemical variables including survey year

	$eGFR_{crea}$	$eGFR_{cysC}$
Survey year (1986–2014)	–0.142***	0
Age	–0.365***	–0.314***
BMI	–0.113***	–0.206***
Waist	–0.082***	–0.243***
Hip	–0.092***	–0.171***
SBP	–0.148***	–0.178***
DBP	–0.131***	–0.171***
Cholesterol	–0.126***	–0.146***
hsCRP	–0.206***	–0.324***
NTproBNP	–0.262***	–0.224***

For peer review only - http://bmjopen.bmj.com/site/about/guidelines.xhtml

$eGFR_{crea}$ and $eGFR_{cysC}$ according to the LM and CAPA formulas respectively and were Ln transformed. *** P <0.001. BMI=body mass index, SBP=systolic blood pressure, DBP=diastolic blood pressure, hsCRP= high sensitivity C–reactive protein, and NTproBNP=N–terminal fragment of the prohormone brain natriuretic peptide.

For peer review only

Table 2. Univariable linear regression analysis between two different estimated glomerular filtration rates (eGFR) and clinical and biochemical variables including survey year and county

	$eGFR_{crea}$		$eGFR_{cysC}$	
	**Unstandardized β**	**Standardized β**	**Unstandardized β**	**Standardized β**
Survey year (1986–2014)	–0.003***	–0.142***	0.000004	–
BMI (kg/m²)	–0.127***	–0.113***	–0.39***	–0.206***
Waist (cm)	–0.104***	–0.082***	–0.515***	–0.243***
Hip (cm)	–0.206***	–0.092***	–0.642***	–0.171***
SBP (mmHg)	–0.19***	–0.148***	–0.384***	–0.178***
DBP (mmHg)	–0.168***	–0.131***	–0.367***	–0.171***
Hypertension (Y/N)†	–0.04***	–0.125***	–0.076***	–0.141***
Diabetes mellitus (Y/N)‡	–0.088***	–0.08***	–0.112***	–0.061***
Active smoker (vs. never smoker)	0.024***	0.058***	–0.043***	–0.061***
Former smoker (vs. never smoker)	–0.013**	–0.033**	–0.023**	–0.035**

For peer review only - http://bmjopen.bmj.com/site/about/guidelines.xhtml

Cholesterol (mmol/L)	–0.105***	–0.126***	–0.204***	–0.146***
hsCRP (mg/L)	–0.032***	–0.206***	–0.085***	–0.324***
NTproBNP (ng/L)	–0.051***	–0.262***	–0.073***	–0.224***
Tertiary education (vs. primary school)	0.01*	0.025*	0.088***	0.124***
Secondary school (vs. primary school)	0.044***	0.115***	0.098***	0.153***
County (1=Västerbotten, 2=Norrbotten)	–0.012***	–0.033***	–0.016**	–0.027**

Unstandardized and standardized β–values are shown and $eGFR_{crea}$ and $eGFR_{cysC}$, respectively, are dependent variables in separate univariable analyses. $eGFR_{crea}$ and $eGFR_{cysC}$ according to the LM and CAPA formulas respectively and were Ln–transformed. * P <0.05, ** P < 0.01, *** P < 0.001. †Systolic blood pressure ≥140 mmHg and/or diastolic blood pressure ≥90 mmHg, and/or antihypertensive medication. ‡ Self–reported and/or use of glucose lowering medication. BMI=body mass index, SBP=systolic blood pressure, DBP=diastolic blood pressure, hsCRP=high sensitivity C–reactive protein, and NTproBNP=N–terminal fragment of the prohormone brain natriuretic peptide.

For peer review only

Table 3. Multivariable linear regression analysis between two different estimated glomerular filtration rates (eGFR) and clinical and biochemical variables including survey year and county

	$eGFR_{crea}$		$eGFR_{cysC}$	
	**Unstandardized β**	**Standardized β**	**Unstandardized β**	**Standardized β**
Survey	–0.004***	–0.187***	–0.001***	–0.037***
BMI (kg/m²)	0.019	0.016	–0.142***	–0.075***
Hypertension (Y/N)†	–0.013***	–0.04***	–0.017**	–0.03**
Diabetes mellitus (Y/N)‡	–0.051***	–0.046***	–0.022	–0.012
Active smoker (vs. never smoker)	0.026***	0.061***	–0.021**	–0.029**
Former smoker (vs. never smoker)	–0.004	–0.011	0.001	0.001
Cholesterol (mmol/L)	–0.119***	–0.143***	–0.129***	–0.092***
hsCRP (mg/L)	–0.023***	–0.15***	–0.064***	–0.246***
NTproBNP (ng/L)	–0.042***	–0.22***	–0.056***	–0.175***

For peer review only

Tertiary education (vs. primary school)	0.007	0.018	0.043***	0.06***
Secondary school (vs. primary school)	0.034***	0.089***	0.053***	0.083***
County (1=Västerbotten, 2=Norrbotten)	–0.006	–0.016	0.003	0.004

Unstandardized and standardized ⍰–values are shown, and eGFRcrea and eGFRcysC, respectively, are dependent variables in separate multivariable analysis. eGFRcrea and eGFRcysC according to the LM and CAPA formulas respectively and were Ln–transformed. * P <0.05, ** P < 0.01, *** P < 0.001. †Systolic blood pressure ≥140 mmHg and/or diastolic blood pressure ≥90 mmHg, and/or antihypertensive medication. ‡ Self–reported and/or use of glucose lowering medication. BMI=body mass index, SBP=systolic blood pressure, DBP=diastolic blood pressure, hsCRP=high sensitivity C–reactive protein, and NTproBNP=N–terminal fragment of the prohormone brain natriuretic peptide.

For peer review only

For peer review only - http://bmjopen.bmj.com/site/about/guidelines.xhtml

Deterioration in renal function in Northern Sweden between 1986 and 2014 – A population–based study

Authorss

Julia de Man Lapidoth[1], Johan Hultdin[2], P. Andreas Jonsson[1], Maria K Svensson[3], Maria Wennberg[4], Tanja Zeller[5], and Stefan Söderberg[1].

Affiliations

1 Department of Public Health and Clinical Medicine, Medicine, Umeå University

2 Department of Medical Biosciences, Clinical Chemistry, Umeå University

3 Department of Medical Sciences, Renal Medicine, Uppsala University

4 Department of Public Health and Clinical Medicine, Sustainable Health, Umeå University

5 University Center of Cardiovascular Science, University Heart and Vascular Center Hamburg

Supplementary tables 1-5

Supplementary table 1. Clinical and biochemical characteristics at each evaluated time–point

Supplementary table 2. Estimated glomerular filtration rate (eGFR) at each time–point

Supplementary table 3. Bivariate correlation analysis (Pearson), survey 2004 excluded

Supplementary table 4. Univariable linear regression analysis, survey 2004 excluded

Supplementary table 5. Multivariable linear regression analysis, survey 2004 excluded

For peer review only

Supplementary Table 1. Clinical and biochemical characteristics at each evaluated time–point

	1986	1990	1994	1999	2004	2009	2014	Missing
N	1604	1535	1537	1432	1489	1390	1198	–
Age	45.7 (45.1–46.2)	45.0 (44.4–45.6)	45.3 (44.7–45.9)	45.5 (44.9–46.1)	45.6 (45.0–46.2)	46.1 (45.5–46.7)	46.6 (45.9–47.2)	0
Female sex (%)	49.4 (46.9–51.8)	50.8 (48.3–53.3)	51.3 (48.8–53.8)	51.5 (48.9–54.1)	51.8 (49.2–54.3)	51.7 (49.0–54.3)	53.0 (50.2–55.8)	0
BMI (kg/m^2)*	25.0 (24.8–25.2)	25.1 (24.9–25.3)	25.5 (25.3–25.7)	25.9 (25.7–26.1)	26.3 (26.1–26.5)	26.3 (26.0–26.5)	26.5 (26.3–26.8)	51
Waist (cm)*	88.5 (88.0 – 89.1)	84.5 (83.9–85.1)	87.0 (86.4–87.7)	88.5 (87.8–89.1)	89.9 (89.3–90.6)	88.0 (87.3–88.7)	88.0 (87.2–88.7)	65
Hip (cm)*	98.0 (97.6 – 98.4)	97.6 (97.2–98.0)	99.4 (99.0–99.8)	102.8 (102.4–103.2)	100.5 (100.0–100.9)	100.8 (100.3–101.3)	98.10 (97.6–98.6)	123
SBP (mmHg)*	127 (126–128)	127 (126–128)	126 (125–127)	128 (127–129)	124 (123–125)	123 (123–124)	120 (119–121)	14
DBP (mmHg)*	80 (79–80)	80 (79–80)	79 (78–79)	79 (78–79)	77 (76–77)	78 (78–79)	79 (79–80)	14
Hypertension (%)†	33.5 (31.2–35.8)	32.3 (29.7–34.9)	31.1 (28.5–33.7)	33.6 (31.1–36.0)	30.1 (26.9–33.3)	34.8 (31.0–38.7)	21.3 (18.6–24.0)	13
Diabetes mellitus (%)‡	3.2 (2.3–4.0)	2.7 (1.9–3.5)	1.9 (1.2–2.6)	2.5 (1.7–3.3)	2.8 (1.9–3.6)	3.0 (2.1–3.8)	3.0 (2.0–4.0)	0
Active smoking (%)	31.7 (29.5–34.0)	31.5 (29.1–33.8)	29.7 (27.5–32.0)	22.3 (20.1–24.4)	21.0 (18.9–23.0)	17.0 (15.0–19.0)	12.9 (11.0–14.8)	40
Former smoking (%)	29.3 (27.1–31.5)	27.8 (25.5–30.0)	26.5 (24.3–28.7)	28.3 (26.0–30.6)	28.7 (26.4–31.1)	28.7 (26.3–31.1)	29.5 (26.9–32.1)	40
Cholesterol (mmol/L)*	6.2 (6.2–6.3)	6.1 (6.0–6.2)	5.9 (5.9–6.0)	5.6 (5.5–5.6)	5.5 (5.5–5.6)	5.4 (5.4–5.5)	5.3 (5.2–5.3)	49
hsCRP (mg/L)*	0.85 (0.80–0.90)	0.92 (0.87–0.98)	1.12 (1.05–1.18)	1.15 (1.08–1.23)	0.85 (0.80–0.90)	0.98 (0.92–1.04)	1.10 (1.03–1.17)	377
NTproBNP (ng/L)*	38.1 (36.3–40.0)	38.0 (36.2–40.0)	43.3 (41.1–45.7)	41.9 (39.8–44.2)	31.5 (29.9–33.2)	38.9 (37.0–41.1)	42.9 (40.9–45.0)	1432
Tertiary education (%)	10.8 (9.3–12.3)	16.4 (14.5–18.2)	20.7 (18.7–22.7)	24.2 (21.9–26.4)	29.1 (26.8–31.4)	32.2 (29.7–34.6)	34.5 (31.8–37.2)	–[1]
Secondary school (%)	22.2 (20.2–24.2)	22.5 (20.4–24.6)	29.3 (27.1–31.6)	35.2 (32.7–37.7)	39.4 (36.9–41.9)	41.0 (38.4–43.6)	48.5 (45.7–51.3)	–[1]
Primary school (%)	62.4 (60.0–64.8)	60.5 (58.1–63.0)	49.5 (47.0–52.0)	40.6 (38.1–43.2)	30.6 (28.3–33.0)	25.3 (23.0–27.5)	15.6 (13.6–17.7)	–[1]

Mean (geometric*) values with 95% confidence intervals are shown. [1] Missing data for education level = 143. †Systolic blood pressure ≥140 mmHg and/or diastolic blood pressure ≥90 mmHg, and/or antihypertensive medication. ‡ Self–reported and/or on glucose lowering medication. Primary school= 1–6 (9) years, and secondary school=7 (10)–12 years. BMI=body mass index, SBP=systolic blood pressure, DBP=diastolic blood pressure, hsCRP= high sensitivity C–reactive protein, NTproBNP=N–terminal fragment of the prohormone brain natriuretic peptide.

For peer review only

For peer review only - http://bmjopen.bmj.com/site/about/guidelines.xhtml

Supplementary Table 2. Estimated glomerular filtration rate (eGFR) at each time–point

	1986	1990	1994	1999	2004	2009	2014	Missing
$eGFR_{crea}$ (LM)								
All	96.3 (95.7–96.9)	94.9 (94.2–95.6)	88.3 (87.5–89.1)	87.6 (86.7–88.5)	101.5 (100.7–102.4)	92.7 (91.7–93.6)	81.6 (80.8–82.5)	370
Men	96.2 (95.3–97.1)	95.8 (94.9–96.8)	89.5 (88.5–90.5)	87.7 (86.5–89.0)	101.6 (100.2–102.9)	93.4 (92.1–94.8)	82.3 (81.2–83.4)	173
Women	96.4 (95.6–97.3)	94.0 (93.0–95.1)	87.1 (86.0–88.3)	87.4 (86.2–88.7)	101.5 (100.4–102.6)	91.9 (90.7–93.2)	81.1 (79.8–82.3)	197
Norrbotten	94.9 (94–95.8)	97.6 (96.6–98.5)	85.5 (84.4–86.6)	86.9 (85.9–87.9)	103.8 (102.5–105.1)	92.0 (90.6–93.5)	80.3 (79.1–81.5)	180
Västerbotten	97.8 (97–98.7)	92.5 (91.5–93.5)	91.4 (90.4–92.4)	88.3 (86.9–89.8)	99.3 (98.2–100.6)	93.4 (92.2–94.6)	83.1 (81.9–84.3)	166
$eGFR_{cysC}$ (CAPA)								
All	100.4 (99.1–101.7)	92.0 (90.9–93.1)	85.8 (84.5–87.1)	88.7 (87.4–90.0)	121.3 (119.3–123.3)	97.2 (95.7–98.7)	84.4 (83.1–85.6)	380
Men	94.4 (92.8–96.0)	88.3 (87.0–89.6)	83.2 (81.5–85.0)	85.0 (83.3–86.7)	115.0 (112.3–117.7)	93.4 (91.5–95.4)	80.4 (78.9–81.8)	176
Women	107.0 (105.1–108.9)	95.6 (93.9–97.4)	88.3 (86.4–90.3)	92.3 (90.4–94.3)	127.4 (124.4–130.4)	100.9 (98.8–103.1)	88.1 (86.2–90.1)	204
Norrbotten	98.4 (96.7–100.2)	94.6 (93.2–96.1)	80.6 (79.3–82.0)	88.8 (87.3–90.4)	124.0 (121.1–127)	96.9 (94.7–99.2)	84.0 (82.3–85.7)	182
Västerbotten	102.5 (100.7–104.4)	89.5 (87.9–91.2)	91.7 (89.4–94.0)	88.4 (86.3–90.6)	118.5 (115.7–121.4)	97.5 (95.7–99.5)	84.8 (83.0–86.6)	174

Data shown are geometric means with 95% confidence intervals for eGFRcrea and eGFRcysC, stratified for year, sex and county (Norrbotten and Västerbotten).

eGFR=estimated glomerular filtration rate (ml/min/1.73 m2), crea=Creatinine, cysC=Cystatin C, LM=Lund–Malmö revised, CAPA= Caucasian, Asian, Paediatric and Adult cohort.

For peer review only - http://bmjopen.bmj.com/site/about/guidelines.xhtml

Supplementary table 3. Bivariate correlation analysis (Pearson), survey 2004 excluded

	$eGFR_{crea}$	$eGFR_{cysC}$
Survey year (1986–2014)	–0.222***	–0.094***
Age	–0.379***	–0.352***
BMI	–0.147***	–0.257***
Waist	–0.112***	–0.297***
Hip	–0.118***	–0.208***
SBP	–0.141***	–0.182***
DBP	–0.121***	–0.167***
Cholesterol	–0.115***	–0.139***
hsCRP	–0.199***	–0.327***
NTproBNP	–0.249***	–0.206***

$eGFR_{crea}$ and $eGFR_{cysC}$ according to the LM and CAPA formulas respectively and were Ln–transformed.

*** P <0.001. BMI=body mass index, SBP=systolic blood pressure, DBP=diastolic blood pressure, hsCRP=high sensitivity C–reactive protein, and NTproBNP=N–terminal fragment of the prohormone brain natriuretic peptide.

For peer review only

For peer review only - http://bmjopen.bmj.com/site/about/guidelines.xhtml

Supplementary table 4. Univariable linear regression analysis, survey 2004 excluded

	$eGFR_{crea}$		$eGFR_{cysC}$	
	**Unstandardized β**	**Standardized β**	**Unstandardized β**	**Standardized β**
Survey year (1986–2014)	−0.004***	−0.222***	−0.003***	−0.094***
Age (years)	−0.006***	−0.379***	−0.008***	−0.352***
Sex (1=male, 2=female)	−0.014***	−0.041***	0.084***	0.153***
BMI (kg/m²)	−0.164***	−0.147***	−0.45***	−0.257***
Waist (cm)	−0.139***	−0.112***	−0.579***	−0.297***
Hip (cm)	−0.259***	−0.118***	−0.715***	−0.208***
SBP (mmHg)	−0.178***	−0.141***	−0.36***	−0.182***
DBP (mmHg)	−0.153***	−0.121***	−0.331***	−0.167***
Hypertension (Y/N)†	−0.043***	−0.131***	−0.079***	−0.155***
Diabetes mellitus (Y/N)‡	−0.103***	−0.095***	−0.123***	−0.072***
Active smoker (vs. never smoker)	0.03***	0.075***	−0.037***	−0.058***
Former smoker (vs. never smoker)	−0.011*	−0.027*	−0.021**	−0.034**
Cholesterol (mmol/L)	−0.094***	−0.115***	−0.178***	−0.139***
hsCRP (mg/L)	−0.03***	−0.199***	−0.078***	−0.327***
NTproBNP (ng/L)	−0.047***	−0.249***	−0.061***	−0.206***
Tertiary education (vs. primary school)	−0.002	−0.005	0.063***	0.096***
Secondary school (vs. primary school)	0.031***	0.083***	0.078***	0.132***
County (1=Västerbotten, 2=Norrbotten)	−0.02***	−0.058***	−0.024***	−0.044***

$eGFR_{crea}$ and $eGFR_{cysC}$ according to the LM and CAPA formulas, respectively and were Ln–transformed. Unstandardized and standardized [illegible]–values are shown and $eGFR_{crea}$ and $eGFR_{cysC}$, respectively, are dependent variables in univariable analyses. * P <0.05, ** P < 0.01, *** P < 0.001. †Systolic blood pressure ≥140 mmHg and/or diastolic blood pressure ≥90 mmHg, and/or antihypertensive medication. ‡ Self–reported and/or use of glucose lowering medication. BMI=body mass index, SBP=systolic blood pressure, DBP=diastolic blood pressure, hsCRP=high sensitivity C–reactive protein, and NTproBNP=N–terminal fragment of the prohormone brain natriuretic peptide.

For peer review only

For peer review only - http://bmjopen.bmj.com/site/about/guidelines.xhtml

Supplementary table 5. Multivariable linear regression analysis, survey 2004 excluded

	$eGFR_{crea}$		$eGFR_{cysC}$	
	**Unstandardized β**	**Standardized β**	**Unstandardized β**	**Standardized β**
Survey	–0.005***	–0.26***	–0.004***	–0.127***
BMI (kg/m²)	–0.01	–0.009	–0.198***	–0.113***
Hypertension (Y/N)†	–0.015***	–0.045***	–0.021***	–0.041***
Diabetes mellitus (Y/N)‡	–0.065***	–0.06***	–0.036*	–0.021*
Active smoker (vs. never smoker)	0.024***	0.06***	–0.028***	–0.044***
Former smoker (vs. never smoker)	–0.003	–0.009	–0.001	–0.001
Cholesterol (mmol/L)	–0.125***	–0.153***	–0.134***	–0.105***
hsCRP (mg/L)	–0.019***	–0.127***	–0.054***	–0.228***
NT–pro–BNP (ng/L)	–0.038***	–0.202***	–0.045***	–0.153***
Tertiary education (vs. primary school)	0.004	0.01	0.037***	0.056***
Secondary school (vs. primary school)	0.03***	0.08***	0.05***	0.085***
County (1=Västerbotten, 2=Norrbotten)	–0.014***	–0.04***	–0.005	–0.009

$eGFR_{crea}$ and $eGFR_{cysC}$ according to the LM and CAPA formulas respectively and were Ln–transformed. Unstandardized and standardized □–values are shown, and $eGFR_{crea}$ and $eGFR_{cysC}$, respectively, are dependent variables in separate multivariable analysis. * P <0.05, ** P < 0.01, *** P < 0.001. †Systolic blood pressure ≥140 mmHg and/or diastolic blood pressure ≥90 mmHg, and/or antihypertensive medication. ‡ Self–reported and/or use of glucose lowering medication. BMI=body mass index, SBP=systolic blood pressure, DBP=diastolic blood pressure, hsCRP=high sensitivity C–reactive protein, and NTproBNP=N–terminal fragment of the prohormone brain natriuretic peptide.

For peer review only

For peer review only - http://bmjopen.bmj.com/site/about/guidelines.xhtml

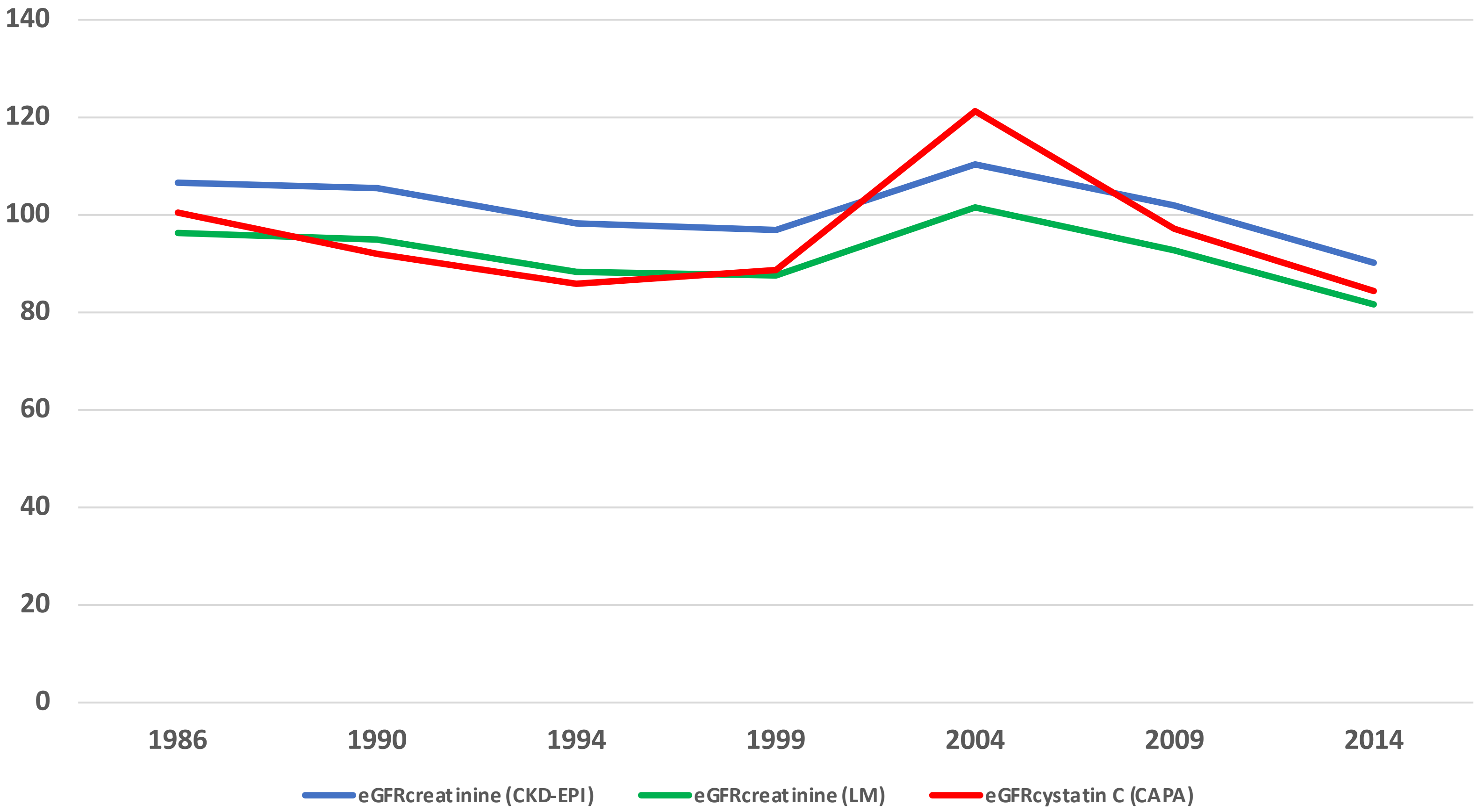
140
120
100
80
60
40
20
0
1986
1990
1994
1999
2004
2009
2014
eGFRcreatinine (CKD-EPI)
eGFRcreatinine (LM)
eGFRcystatin C (CAPA)

STROBE 2007 (v4) Statement—Checklist of items that should be included in reports of *cross-sectional studies*

Section/Topic	Item #	Recommendation	Reported on page #
**Title and abstract**	1	(*a*) Indicate the study's design with a commonly used term in the title or the abstract	2
		(*b*) Provide in the abstract an informative and balanced summary of what was done and what was found	2
**Introduction**			
Background/rationale	2	Explain the scientific background and rationale for the investigation being reported	6-7
Objectives	3	State specific objectives, including any prespecified hypotheses	7
**Methods**			
Study design	4	Present key elements of study design early in the paper	7
Setting	5	Describe the setting, locations, and relevant dates, including periods of recruitment, exposure, follow-up, and data collection	7
Participants	6	(*a*) Give the eligibility criteria, and the sources and methods of selection of participants	7
Variables	7	Clearly define all outcomes, exposures, predictors, potential confounders, and effect modifiers. Give diagnostic criteria, if applicable	8-9
Data sources/ measurement	8*	For each variable of interest, give sources of data and details of methods of assessment (measurement). Describe comparability of assessment methods if there is more than one group	8-10
Bias	9	Describe any efforts to address potential sources of bias	12
Study size	10	Explain how the study size was arrived at	7, 11
Quantitative variables	11	Explain how quantitative variables were handled in the analyses. If applicable, describe which groupings were chosen and why	10
Statistical methods	12	(*a*) Describe all statistical methods, including those used to control for confounding	10
		(*b*) Describe any methods used to examine subgroups and interactions	12-13
		(*c*) Explain how missing data were addressed	11 and Table 1
		(*d*) If applicable, describe analytical methods taking account of sampling strategy	NA
		(*e*) Describe any sensitivity analyses	11
**Results**			

For peer review only

Participants	13*	(a) Report numbers of individuals at each stage of study—eg numbers potentially eligible, examined for eligibility, confirmed eligible, included in the study, completing follow-up, and analysed	11, table 1
		(b) Give reasons for non-participation at each stage	NA
		(c) Consider use of a flow diagram	NA
Descriptive data	14*	(a) Give characteristics of study participants (eg demographic, clinical, social) and information on exposures and potential confounders	Table 1
		(b) Indicate number of participants with missing data for each variable of interest	Table 1
Outcome data	15*	Report numbers of outcome events or summary measures	12, table 2
Main results	16	(*a*) Give unadjusted estimates and, if applicable, confounder-adjusted estimates and their precision (eg, 95% confidence interval). Make clear which confounders were adjusted for and why they were included	Table 2
		(*b*) Report category boundaries when continuous variables were categorized	Table 1 and 2
		(*c*) If relevant, consider translating estimates of relative risk into absolute risk for a meaningful time period	NA
Other analyses	17	Report other analyses done—eg analyses of subgroups and interactions, and sensitivity analyses	See above, 11
**Discussion**			
Key results	18	Summarise key results with reference to study objectives	13
Limitations	19	Discuss limitations of the study, taking into account sources of potential bias or imprecision. Discuss both direction and magnitude of any potential bias	15-16
Interpretation	20	Give a cautious overall interpretation of results considering objectives, limitations, multiplicity of analyses, results from similar studies, and other relevant evidence	15-16
Generalisability	21	Discuss the generalisability (external validity) of the study results	16-17
**Other information**			
Funding	22	Give the source of funding and the role of the funders for the present study and, if applicable, for the original study on which the present article is based	4

*Give information separately for cases and controls in case-control studies and, if applicable, for exposed and unexposed groups in cohort and cross-sectional studies.

Note: An Explanation and Elaboration article discusses each checklist item and gives methodological background and published examples of transparent reporting. The STROBE checklist is best used in conjunction with this article (freely available on the Web sites of PLoS Medicine at http://www.plosmedicine.org/, Annals of Internal Medicine at http://www.annals.org/, and Epidemiology at http://www.epidem.com/). Information on the STROBE Initiative is available at www.strobe-statement.org.

For peer review only

BMJ Open

Trends in renal function in Northern Sweden 1986 – 2014: data from the seven cross-sectional surveys within the Northern Sweden MONICA study

Journal:	*BMJ Open*
Manuscript ID	bmjopen-2023-072664.R1
Article Type:	Original research
Date Submitted by the Author:	16-Jun-2023
Complete List of Authors:	de Man Lapidoth, Julia; Umeå Universitet Medicinska fakulteten, Dept of Public Health and Clinical Medicine<br>Hultdin, Johan; Umeå University, Department of Medical Biosciences, Clinical Chemistry<br>Jonsson, P. Andreas; Umeå Universitet Medicinska fakulteten, Dept of Public Health and Clinical Medicine<br>Eriksson Svensson, Maria; Uppsala University, Renal medicine<br>Wennberg, Maria; Umeå University, Department of Public Health and Clinical Medicine<br>Zeller, Tanja ; University of Hamburg, Clinic for general and interventional Cardiology<br>Söderberg, Stefan ; Umeå University, Department of Public Health and Clinical Medicine
**Primary Subject Heading**:	Renal medicine
Secondary Subject Heading:	Epidemiology, Public health
Keywords:	EPIDEMIOLOGY, PUBLIC HEALTH, NEPHROLOGY

SCHOLARONE™
Manuscripts

I, the Submitting Author has the right to grant and does grant on behalf of all authors of the Work (as defined in the below author licence), an exclusive licence and/or a non-exclusive licence for contributions from authors who are: i) UK Crown employees; ii) where BMJ has agreed a CC-BY licence shall apply, and/or iii) in accordance with the terms applicable for US Federal Government officers or employees acting as part of their official duties; on a worldwide, perpetual, irrevocable, royalty-free basis to BMJ Publishing Group Ltd ("BMJ") its licensees and where the relevant Journal is co-owned by BMJ to the co-owners of the Journal, to publish the Work in this journal and any other BMJ products and to exploit all rights, as set out in our licence.

The Submitting Author accepts and understands that any supply made under these terms is made by BMJ to the Submitting Author unless you are acting as an employee on behalf of your employer or a postgraduate student of an affiliated institution which is paying any applicable article publishing charge ("APC") for Open Access articles. Where the Submitting Author wishes to make the Work available on an Open Access basis (and intends to pay the relevant APC), the terms of reuse of such Open Access shall be governed by a Creative Commons licence – details of these licences and which Creative Commons licence will apply to this Work are set out in our licence referred to above.

Other than as permitted in any relevant BMJ Author's Self Archiving Policies, I confirm this Work has not been accepted for publication elsewhere, is not being considered for publication elsewhere and does not duplicate material already published. I confirm all authors consent to publication of this Work and authorise the granting of this licence.

For peer review only

Trends in renal function in Northern Sweden 1986 – 2014: data from the seven cross-sectional surveys within the Northern Sweden MONICA study

Short title: Trends in renal function over time

Authors list:

Julia de Man Lapidoth[1], Johan Hultdin[2], P. Andreas Jonsson[1], Maria K Svensson[3], Maria Wennberg[4], Tanja Zeller[5,6], and Stefan Söderberg[1].

Affiliations:

[1]Department of Public Health and Clinical Medicine, Medicine, Umeå University

[2]Department of Medical Biosciences, Clinical Chemistry, Umeå University

[3]Department of Medical Sciences, Renal Medicine, Uppsala University

[4]Department of Public Health and Clinical Medicine, Sustainable Health, Umeå University

[5]University Center of Cardiovascular Science, University Heart and Vascular Center Hamburg

[6] German Center of Cardiovascular Research (DZHK), Partner Seite Hamburg, Kiel, Lübeck, Hamburg/Germany

Correspondence: Stefan Söderberg, Department of Public Health and Clinical Medicine, Medicine, Umeå University. Phone: +46 70 319 38 03, email: stefan.soderberg@umu.se

Word count and supplementary material

Main manuscript – 3935 (except references and tables), abstract – 245, references – 48, and tables – 3, figure - 1.

Supplementary material: 5 tables.

For peer review only

Abstract:

Objective: The prevalence of chronic kidney disease (CKD) is increasing globally, and CKD is closely related to cardiovascular disease (CVD). CKD and CVD share several risk factors (RF), such as diabetes, hypertension, obesity, and smoking, and the prevalence of these RF has changed during the last decades, and we aimed to study the effect on renal function over time.

Design: Repeated cross-sectional population-based studies

Setting: The two Northern counties (Norr- and Västerbotten) in Sweden.

Participants: Within the MONitoring Trends and Determinants of CArdiovascular Disease (MONICA) study, seven surveys were performed between 1986 and 2014, including participants aged 25 to 64 years (n=10,185).

Interventions: None

Measures: Information on anthropometry, blood pressure and cardiovascular risk factors was collected. Creatinine and cystatin C were analyzed in stored blood samples and the estimated glomerular filtration rate (eGFR) calculated using the creatinine-based Lund–Malmö (LM) revised and Chronic Kidney Disease Epidemiology Collaboration (CKD–EPI) ($eGFR_{crea}$) equations, as well as the cystatin C-based Caucasian, Asian, Paediatric and Adult cohort (CAPA) equation ($eGFR_{cysC}$). Renal function over time was analyzed using uni– and multivariable linear regression models.

Results: Renal function, both $eGFR_{crea}$ and $eGFR_{cysC,}$ decreased over time (both $P < 0.001$), and differed between counties and sexes. In a multivariable analysis, study year remained

For peer review only - http://bmjopen.bmj.com/site/about/guidelines.xhtml

inversely associated with both $eGFR_{crea}$ and $eGFR_{cysC}$ (both $P<0.001$) after adjustment for classical cardiovascular RF.

Conclusion: Renal function has deteriorated in Northern Sweden between 1986 – 2014.

Key words: estimated glomerular filtration rate; renal function; creatinine; cystatin C; cardiovascular risk factors

For peer review only

Strengths and limitations of this study

- Study design, selection of participants and measurements consistent over time.
- Population-based study.
- Creatinine and Cystatin C measured in the same laboratory during the same period.
- Several risk factors were self-reported.
- Possible residual confounding.

For peer review only

Introduction

The prevalence of chronic kidney disease (CKD) is increasing globally, and deaths attributed to CKD constituted 4.6% of all deaths in 2017.[1] According to the CaReMe study, 8.3 % of the population in Sweden has CKD defined as having a diagnosis, an estimated glomerular filtration rate (eGFR) < 60 ml/min/1.73m^2 or urine albumin-creatinine ratio (UACR) ≥ 30 mg/g.[2] Furthermore, medical treatment due to end-stage kidney disease (ESKD) is costly and constitutes approximately 1% of the total medical expenditures in Sweden.[3]

Decreased renal function is associated with an increased risk for cardiovascular events, including cardiovascular death.[4,5] This is partly due to shared risk factors for CKD and cardiovascular disease (CVD), for example diabetes, hypertension, obesity, and smoking.[6-8] Other possible mechanisms such as systemic inflammation, renal anemia, and endothelial cell dysfunction may contribute.[9] In addition, data suggest that subjects with CVD and CKD get less secondary prevention after a cardiovascular event.[10,11]

In Northern Sweden, the prevalence of hypertension, smoking, and hypercholesterolemia have decreased, whereas obesity has increased.[12] In parallel, the use of antihypertensive drugs has increased.[12] Furthermore, low socioeconomic status is associated with decreased renal function, possibly due to the clustering of other cardiovascular risk factors.[13] Longitudinally, there are thus several factors that might have impacted renal function both negatively and positively over time, and the potential net effect is not known.

This study aimed to investigate the development on renal function over 28 years in Northern Sweden, considering the concomitant changes in the prevalence of major cardiovascular risk factors and other factors that may impact renal function over time.

For peer review only - http://bmjopen.bmj.com/site/about/guidelines.xhtml

Methods

This study is based on seven cross-sectional surveys performed within the framework of the Northern Sweden MONICA (multinational MONItoring of trends and determinants in CArdiovascular disease) study.[14] The World Health Organizations' MONICA project was initiated in the mid–eighties, and the first survey in the two most northern counties of Sweden (Västerbotten and Norrbotten) was performed in 1986. Altogether seven population–based cross sectional surveys using similar methods have been conducted (in 1986, 1990, 1994, 1999, 2004, 2009, and 2014). All surveys were performed during the same months (January – April) to eliminate seasonal variations. The participants have been randomly selected to represent the population and make the different surveys comparable. Two examination teams collected all data using standardized methods to the extent it is possible. Participants were invited if aged between 25–64 years in 1986 and 1990 (n=2,000 per survey), and 25–74 years from 1994 (n=2,500 per survey). Altogether 11,924 subjects participated in seven surveys with a declining participation rate at each survey (mean 72 %, range 81–61%). The majority of participants were Swedish Caucasians, with a small minority of Finnish and Sami heritage.

Patient and public involvement

The public or any patient organisations were not involved in study design, analysis of data or in preparing the manuscript.

Questionnaires

All participants answered questions about diabetes mellitus (yes/no/don't know). Those who answered yes or had diabetes medications were considered as having diabetes. Questions regarding smoking involve active smoking, sporadic smoking, former smoking, former

For peer review only - http://bmjopen.bmj.com/site/about/guidelines.xhtml

sporadic smoking, and non–smoker. The answers were recoded into active smoking (active and sporadic smokers), former smoking (former and former sporadic smokers), and non–smoker. Medications taken regularly were grouped according to ATC–code. Participants who reported treatment with angiotensin–converting enzyme inhibitor (ACEi), angiotensin receptor blocker (ARB) or mineralocorticoid receptor antagonist (MRA) were identified. These treatments were summarized as treatment with a renin-angiotensin-aldosterone (RAAS) inhibitor.

The self-reported level of attained education was categorized into tertiary education (university), secondary school, and primary school.

Measurements

Height was measured without shoes using a calibrated scale with a measuring rod attached to the scale or mounted on the wall. The participants wore thin clothing to measure their weight, and a calibrated scale was used (a balance scale 1986–1994, from 1999, an electrical scale was used). The weight was estimated to nearest 0.2 kilograms. Body mass index was calculated as weight divided by height squared.[15] The waist and hip circumference were measured with a tape measure without clothes between the iliac crest and the lowest rib, over the symphysis bone, and over the part of the hips with the greatest width. With this information, the waist–hip ratio was calculated.[15] After five minutes of rest in the sitting position, blood pressure was measured in the right arm twice at a three-minute interval, and the mean value was calculated. The Hawksley random–zero sphygmomanometer was used between 1986 and 2004, and from 2009 the Omron M7–device was used.[16] Hypertension was defined as systolic blood pressure ≥140 mmHg, diastolic blood pressure ≥ 90 mmHg, or the use of antihypertension medication for at least two weeks before the survey.

For peer review only - http://bmjopen.bmj.com/site/about/guidelines.xhtml

Blood samples

Blood samples were drawn after fasting for at least 4 hours. Total cholesterol levels were determined at a regional laboratory within 24 hours using an enzymatic method in 1986–1994. From 1999, a dry–chemistry method was used.[17]

In 2015, stored serum samples from all surveys (stored frozen at –80° C) were sent to Hamburg/Germany, as part of the BiomarCaREproject, for further analysis of Cystatin C, Creatinine, N–terminal fragment of the prohormone brain natriuretic peptide (NTproBNP), and high-sensitivity C-reactive protein (hsCRP).[18] For approximately 50% of the samples from 1990, etheylenediaminetetraacetic acid (EDTA) plasma was used instead of serum.

Cystatin C was analyzed using a latex immunoturbidimetric assay and creatinine with a Jaffe / kinetic assay (Kinetic Alkaline Picrate), performed on an Architect C8000 system (Abbott, Abbot Park, IL, USA). Measurement ranges were for creatinine 17.7–3271 µmol/L with a limit of quantification (LOQ) of 4.4 µmol/L. Creatinine was IDMS (isotope dilution mass spectrometry) traceable (NIST SRM 967). Cystatin C had a measurement range of 0.05 – 9.6 mg/L, and the LOQ was 0.05 mg/L. hsCRP was analyzed with a highly sensitive latex immunoturbidimetric assay. NTproBNP was measured using Electrochemiluminescence sandwich immunoassay ECLIA on an ELECSYS 2010 platform (Roche Diagnostics, Basel, Switzerland).

In addition, in 2009 and 2014 creatinine was measured locally with an IDMS traceable method.

Statistical analyses

For peer review only

Two different formulas for calculating the creatinine–based eGFR ($eGFR_{crea}$) were used, the Lund–Malmö (LM) revised formula[19] and the Chronic Kidney Disease Epidemiology Collaboration (CKD–EPI) formula from 2009.[20] To estimate cystatin C–based eGFR ($eGFR_{cysC}$), the Caucasian, Asian, Paediatric, and Adult cohort (CAPA) formula[21] was used. For the CKD–EPI calculation, all participants were labeled as Caucasians.

- LM revised formula: $e^{X - 0.0158 \times Age + 0.438 \times \ln(Age)}$, if female and creatinine <150 X = 2.50 + 0.0121 × (150–creatinine), if female and creatinine ≥150 X = 2.50 – 0.926 × Ln(creatinine/150), if male and creatinine < 180 X = 2.56 + 0.00926 × (180 – creatinine), if male and creatinine ≥ 180 X = 2.56 – 0.926 × Ln(creatinine/180)
- CKD–EPI formula from 2009: $141 \times \min\left(\frac{Scr}{\kappa, 1}\right)^{\alpha} \times \max\left(\frac{Scr}{\kappa, 1}\right)^{-1.209} \times 0.993^{Age}$ $\times 1.018$ [if female] $\times 1.159$ [if black], *Scr* = serum creatinine, *κ* = 0.7 (male) and 0.9 (female), *α* = –0.411 (male) and –0.329 (female), min = the minimum of *Scr*/ *κ* or 1 and max = the maximum of *Scr*/*κ* or 1.
- CAPA = $130 \times (cystatin\ C^{-1.069}) \times (age^{-0.117})$ –7

Using the Dubois–Dubois formula,[22] absolute eGFR was also calculated.

$$\frac{eGFR \times (0.007184 \times length^{0.725} \times weight^{0.425}}{1.73}$$

Persons aged 25 to 64.9 years were included in the analysis. Descriptive data included mean values with 95% confidence intervals and medians with interquartile ranges. If necessary, some variables were ln–transformed to improve normality, and geometric means were calculated. Pearson's correlation analyses were performed. To evaluate associations between eGFR and year of survey adjusted for major cardiovascular risk factors, uni– and multivariable linear regression analyses were performed.

For peer review only - http://bmjopen.bmj.com/site/about/guidelines.xhtml

Plasma concentrations of creatinine and cystatin C were markedly higher in 2004. Therefore, both results including and excluding data from 2004 are presented.

Most continuous variables were ln–transformed to improve normality. Number of missing data are reported in Table 1 and no imputation was done.

To test the robustness of the results, hsCRP and NTproBNP and data from 2004 were excluded from the multivariable model in separate steps, and different formulas for calculation of eGFR were used.

Age and sex were not included in the linear regression or correlation analyses, as these variables are included in the equations of eGFR. However, as a sensitivity analysis, we added age to the multivariable analysis.

For categorical variables with more than two groups, dummy variables were created, including a separate dummy variable for missing data which was omitted from the tables.

The SPSS statistical program version 28 was used (IBM Corporation, New York, NY, USA).

Results

Baseline data from 7 surveys are presented in Table 1. The study included 10,185 participants with a mean age (95% confidence interval) of 46 (24–65) years, and 51% were women.

The prevalence of major cardiovascular risk factors changed between 1986 and 2014: total cholesterol, systolic blood pressure, and active smoking decreased, whereas BMI increased. hsCRP, NTproBNP, and the proportion of the participants using drugs interacting with the

For peer review only

RAAS system (ACEi, ARB, and MRA) increased (0 to 10.2 %). A notable increase in the proportion of participants with higher education, was seen over 28 years.

We found that $eGFR_{crea}$ (both revised LM and the CKD–EPI) and $eGFR_{cysC}$ (CAPA) decreased over time (Table 2 and Figure 1). In line with this, the percentage of participants with an eGFR below 60 ml/min/1.73 m^2 increased. Overall, a more pronounced decrease in renal function (eGFR) was observed over time in Norrbotten county compared to Västerbotten county. Women had higher $eGFR_{cysC}$, but a lower $eGFR_{crea}$ compared to men. The $eGFR_{crea}$ measured locally was lower in 2014 compared to 2009, 84.0 (83.3 – 84.7) versus 84.8 (84.2 – 85.4), P=0.03.

In the correlation analysis, study year associated inversely with $eGFR_{crea}$ (Supplementary table 1, *P*=<0.001), and with both $eGFR_{crea}$ and $eGFR_{cysC}$ if data from 2004 were excluded (Supplementary Table 2, *P*=<0.001). Age, anthropometric measures, blood pressure, total cholesterol, and CRP and NTproBNP were inversely associated with both $eGFR_{crea}$ and $eGFR_{cysC}$ irrespectively if data from 2004 were included or not.

The univariable linear regression analyses displayed the same pattern (Supplementary Table 3 and 4). Study year associated inversely with $eGFR_{crea}$(*P*=<0.001) and with both $eGFR_{crea}$ and $eGFR_{cysC}$ if data from 2004 were excluded (*P*=<0.001). Furthermore, female sex associated with lower $eGFR_{crea}$ (*P*=0.001) but with higher $eGFR_{cysC}$ (*P*=<0.001). Higher attained educational level associated with higher $eGFR_{crea}$ and higher $eGFR_{cysC}$. Active smokers had higher $eGFR_{crea}$ but lower $eGFR_{cysC}$. These results based on the LM and CAPA formulas were almost identical if the CKD–EPI formula was used instead or if absolute eGFR was calculated (data not shown).

For peer review only - http://bmjopen.bmj.com/site/about/guidelines.xhtml

In the final multivariable linear regression models, study year remained inversely associated with both $eGFR_{crea}$ and $eGFR_{cysC}$ (Table 3, both *P*=<0.001). This was seen irrespective of including or excluding hsCRP and NTproBNP (data not shown) or data from 2004 (Supplementary Table 5). Survey year remained inversely associated with $eGFR_{crea}$ and $eGFR_{cysC}$ if age was included in the multivariable model (data not shown). Lower $eGFR_{crea}$ associated with diabetes, hypertension, and higher cholesterol, and with higher CRP and NTproBNP levels, and lower $eGFR_{cysC}$ associated with increasing BMI, hypertension, higher cholesterol, active smoking, and with higher CRP and NTproBNP levels. In contrast, active smoking associated with higher $eGFR_{crea}$. A higher educational level remained positively associated with both $eGFR_{crea}$ and $eGFR_{cysC}$, whereas county did not associate with eGFR after adjustments. Using the CKD–EPI equation gave almost identical results (data not shown).

Study year remained inversely associated with $eGFR_{crea}$ and $eGFR_{cysC}$ if hypertension was replaced with systolic and diastolic blood pressure, if the use of ACEi, ARB, and MRA was added, or if the waist and hip circumferences were added (data not shown). High blood pressure and the use of RAAS blocking agents (ACEi, ARB, and MRA) were all independently associated with lower $eGFR_{crea}$ and $eGFR_{cysC}$ (data not shown).

For peer review only

Discussion

In this study we found that renal function assessed as $eGFR_{crea}$ and $eGFR_{cysC}$ have decreased over 28 years in Northern Sweden. In parallell, the proportion of persons with aeGFR below 60 ml/min/1.73 m^2 increased. This was also seen after adjustment for concomitant changes in the prevalence of major cardiovascular risk factors and other known cardiovascular risk

For peer review only - http://bmjopen.bmj.com/site/about/guidelines.xhtml

factors that may impact renal function over time. Notably, the renal function differed between the studied counties, possibly due to varying cardiovascular risk profiles, as the difference did not remain after adjustments. Possibly, the ongoing primary–care intervention program in Västerbotten may have had an impact.[23] We also found that women have higher $eGFR_{cysC}$ but lower $eGFR_{crea}$ than men, which is not in line with earlier studies showing that male gender is usually associated with a lower $eGFR_{crea.}$[24] These results are in line with increasing prevalence of end-stage renal disease (ESRD) between 1990 and 2021 in Sweden.[25] The incidence is also increasing but not as evident as the prevalence, probably due to better care and longer survival. In contrast, the prevalence of CKD not requiring replacement therapy is largely unknown in Sweden as these alterations are not uniformly registered. The CaReMe-study showed that approximately 8.3% of the Swedish population has CKD.[2]

To our knowledge, a similar study of renal function with repeated measurements of both creatinine and cystatin C over a long time has not been done previously. Several studies have focused on changes in renal function in the same cohort over time, showing deteriorating renal function with increasing age.[24] Two previous population-based studies in Scandinavia have followed renal function over time.[26,27] They were mainly based on creatinine measurements and covered shorter periods of time. In Norway, the prevalence of CKD was stable for more than a decade while the eGFR decreased in Finland over 5 years.

We adjusted for several factors to try to explain the deteriorating renal function. However, the decline in eGFR remained an independent finding in the multivariable analysis. This finding may be worrying as reduced renal function is associated with an increased mortality risk.[28] Hypertension and smoking contribute to a faster decline in renal function,[7] and

For peer review only - http://bmjopen.bmj.com/site/about/guidelines.xhtml

the use of antihypertensive medications has increased in northern Sweden,[29] including RAAS–inhibitors ACEi, ARB, and MRA. These medications are known to initially decrease the eGFR due to a reduction of glomerular hyperfiltration[30] and could partly explain an observed decrease in renal function over time. The observed inverse relationship between the use of RAAS–inhibitors could also be due to co–linearity with the conditions *(i.e.* hypertension and renal protection) that they are used for. However, the long–term treatment with these medications preserve renal function.[31]

According to a previous study on this population, the reported percentage of energy intake from protein increased slightly from 14 to 16 % from 1994 until 2014. It is unlikely that this small increase had any large impact on the finding of increasing creatinine.[32] In addition, increasing creatinine and a lower estimated eGFR is further supported by the finding of decreasing renal function when using cystatin C to estimate renal function.

Another potential explanation could be increased exposure to environmental pollutants,[33] but previous studies have shown a decrease in lead in the population and a stable amount of cadmium.[34] However, there are numerous other known and unknown pollutants that could potentially affect renal function.

Northern Sweden also has a high incidence and prevalence of the viral infection nephropathia epidemica (Puumala virus),[35] which is a hemorrhagic fever with a renal syndrome (HFRS). The long–term effects are still unknown, and earlier studies have shown contradictory results if the infection causes irreversible long-term kidney damage.[36,37]

Smoking affected $eGFR_{crea}$ and $eGFR_{cysC}$ differentially, which has been presented earlier.[38] It has been suggested that smoking causes glomerular hyperfiltration[39] affecting creatinine more but also that sarcopenia may contribute to lower creatinine values in

For peer review only - http://bmjopen.bmj.com/site/about/guidelines.xhtml

smokers. Possibly, smoking per se may increase cystatin C levels[40] which is supported by studies showing a decrease in cystatin C following smoking cessation.[41]

This study shows that higher education is associated with better renal function, which is in line with previous studies.[42] Lower socioeconomic status (SES) is associated with a higher incidence of CKD,[43] and a higher risk of chronic renal failure.[44] Several indicators for SES have been used, such as educational attainment, household, or individual income, or geocoordinates. However, similar results related to renal function have been shown irrespective of how SES has been defined.[42]

Strengths and limitations

The strength of this study is that renal function is measured using two different endogenous markers, creatinine and cystatin C, which are affected by different confounding factors[45,46] and both endogenous markers show the same trend of decreasing renal function. An additional strength is that data is collected from a randomly selected, large cohort, representing the general population at the time of each survey, which makes the results applicable to the general population. One additional strength is that all creatinine and cystatin C analyses for the different years were performed within the same period, at the same laboratory, using the same methods.

There are several limitations as well. Some of the blood samples have been frozen for a long period, the earliest from 1986, which could affect the results of the biochemical analyses. Prolonged storage time could result in the degradation of biomarkers and sublimation of water from the frozen samples altering concentrations of the biomarkers. Still, the finding of a reduction of both $eGFR_{crea}$ and $eGFR_{cysC}$ over time suggests that there is an actual reduction in renal function (eGFR). Furthermore, it should be emphasized that the samples have been

For peer review only - http://bmjopen.bmj.com/site/about/guidelines.xhtml

stored in –80° degrees from sampling to analysis without thawing. The method to analyze creatinine, the Jaffe/kinetic assay, is not specific and other compounds (for example, hemoglobin F, certain proteins, glucose, and ketone bodies) produce Jaffe–like chromogens (also known as pseudocreatinines or non–creatinine chromogens). To adjust for this potential overestimation, compensated analyses can be used. Since all blood samples are analyzed using the same method, it is possible to use this method to investigate renal function over time in the population. However, we saw variations in levels of biomarkers related to survey year, the 2004 results in particular. We therefore repeated all analyses after excluding 2004 data which gave similar results. Quality indicates have been scrutinized, but no obvious reason has been found for this discrepancy.

Other limitations lay in that some of the data included in this study are self–reported (diabetes, smoking, medication), which makes recall bias impossible to eliminate. The participation rate has been very good in the MONICA surveys (72%), which is high compared to other population–based surveys internationally. There is always a selection in this kind of studies, and healthier and older individuals are more likely to participate. Since eGFR is an estimation of renal function and not a direct measurement, it is possible that these results are not exclusively based on a decreasing renal function but could also be affected by changes in living conditions and measurements. One known risk factor is alcohol intake.[47] This is not included in this study due to the lack of reliability of self–reported alcohol intake but a previous study on dietary trends in this population found an increase in reported energy from alcohol over time.[32] We also lack information about medications bought over the counter (OTC) such as non–steroidal anti–inflammatory drug (NSAID) that may impact renal function both short term and long-term.[48] Finally, the results are based on a

For peer review only - http://bmjopen.bmj.com/site/about/guidelines.xhtml

For peer review only

predominately Caucasian population in Northern Scandinavia, and the generalizability to other ethnicities and contexts is unknown.

Conclusion

In this study we found that estimated renal function have deteriorated in Northern Sweden independently of changes in cardiovascular risk factors and socioeconomic status.

For peer review only

For peer review only - http://bmjopen.bmj.com/site/about/guidelines.xhtml

Acknowledgement

The authors thank the MONICA project, scientific leaders, and participants through the years, as well as the Department of Biobank Research at Umeå University (https://www.umu.se/en/biobank–research–unit/) for providing data and blood samples.

The authors also want to thank the MORGAM/BiomarCaRe team in Hamburg and Helsinki for the extensive analyses of biomarkers. A special thanks to Professor Kari Kuulasma in Helsinki, Finland and to Dr Stefan Blankenberg in Hamburg, Germany.

For peer review only

Figure legend

Renal function in Northern Sweden between 1986 and 2014 expressed as eGFR (geometric mean) based on the Lund–Malmö (LM) revised formula and the Chronic Kidney Disease Epidemiology Collaboration (CKD–EPI) formula based on creatinine, and the Caucasian, Asian, Paediatric, and Adult cohort (CAPA) based on Cystatin C.

For peer review only

Contributorship statement

SS is principal investigator for the Northern Sweden MONICA study and is responsible for the repeated population-based surveys and the collection of data and samples. SS initiated this study and drafted the main hypothesis. TZ is responsible for the analyses of Creatinine and Cystatin C and related qualitative checks. JDL made the primary analyses and drafted the manuscript. JH, PAJ, MKS and MW have together with JDL conducted literature search and prepared the manuscript. JDL, SS, JH, PAJ, MKS, MW and TZ have all been responsible for carefully interpretation of results, contribution to and revision of the manuscript, and they have read and approved the final version of the manuscript, as well as agreed to be accountable for all aspects of the work. SS and JDL have full access to the dataset.

(Abbreviations: JDL for Julia de Man Lapidoth, JH for Johan Hultdin, PAJ for P. Andreas Jonsson, MKS for Maria K Svensson, MW for Maria Wennberg, TZ for Tanja Zeller, and SS for Stefan Söderberg.)

Competing interests' statement

MKS reports no conflict of interest or competing interests to declare related to this project. Provided expertise in ad boards and given lectures for Amgen, AstraZeneca, Boehringer Ingelheim and GSK. Been a clinical trialist and scientific collaborations with Bayer, MSD, Novo Nordisk, AstraZeneca and Vifor Pharma. SS reports speaker honoraria from Actelion Ltd. All others declare no disclosures.

Funding

The Northern Sweden MONICA study was funded by the county councils in Norr– and Västerbotten, and by Umeå university. The BiomarCaRE Project was funded by the European Union Seventh Framework Programme (FP7/2007–2013) under grant agreement No.

For peer review only

HEALTH–F2–2011–278913. Dr Lapidoth was supported by ALF from the county council of Västerbotten.

Data sharing statement

Data from the Northern MONICA surveys are not freely available according to GDPR due to the presence of individual data. However, pseudonymized data can be shared after ethical evaluation and formal request (https://www.umu.se/en/biobankresearch–unit/research/access–to–samples–anddata/access–to–nsdd/).

Ethical approval statement

All participants have signed informed consent for future research using collected data and blood samples. The Ethics Review Board in Umeå has approved the study protocol according to the Declaration of Helsinki (for surveys 2013/97–31 and for biochemical analyses 2012–280–32M).

For peer review only

For peer review only - http://bmjopen.bmj.com/site/about/guidelines.xhtml

References

1. Purcell CA, Abebe M, Adebayo OM, Afarideh M, Agarwal SK, Agudelo-Botero M, Alipour V, Al-Raddadi RM, Alvis-Guzman N, Andualem Z, et al. Global, regional, and national burden of chronic kidney disease, 1990–2017: a systematic analysis for the Global Burden of Disease Study 2017. *The Lancet (British edition)*. 2020;395:709-733. doi: 10.1016/S0140-6736(20)30045-3
2. Sundstrom J, Bodegard J, Bollmann A, Vervloet MG, Mark PB, Karasik A, Taveira-Gomes T, Botana M, Birkeland K, Thuresson M, et al. Prevalence, outcomes, and cost of chronic kidney disease in a contemporary population of 2.4 million patients from 11 countries: The CaReMe CKD study. *The Lancet regional health Europe*. 2022;20. doi: 10.1016/j.lanepe.2022.100438
3. Eriksson JK, Neovius M, Jacobson SH, Elinder CG, Hylander B. Healthcare costs in chronic kidney disease and renal replacement therapy: a population-based cohort study in Sweden. *BMJ Open*. 2016;6:e012062. doi: 10.1136/bmjopen-2016-012062
4. Rangaswami J, Bhalla V, Blair JEA, Chang TI, Costa S, Lentine KL, Lerma EV, Mezue K, Molitch M, Mullens W, et al. Cardiorenal Syndrome: Classification, Pathophysiology, Diagnosis, and Treatment Strategies: A Scientific Statement From the American Heart Association. *Circulation (New York, NY)*. 2019;139:e840-e878. doi: 10.1161/CIR.0000000000000664
5. Go AS, Chertow GM, Fan D, McCulloch CE, Hsu CY. Chronic kidney disease and the risks of death, cardiovascular events, and hospitalization. *N Engl J Med*. 2004;351:1296-1305. doi: 10.1056/NEJMoa041031
6. Tuttle KR, Alicic RZ, Duru OK, Jones CR, Daratha KB, Nicholas SB, McPherson SM, Neumiller JJ, Bell DS, Mangione CM, et al. Clinical Characteristics of and Risk Factors for Chronic Kidney Disease Among Adults and Children: An Analysis of the CURE-CKD Registry. *JAMA network open*. 2019;2:e1918169. doi: 10.1001/jamanetworkopen.2019.18169
7. Toyama T, Kitagawa K, Oshima M, Kitajima S, Hara A, Iwata Y, Sakai N, Shimizu M, Hashiba A, Furuichi K, et al. Age differences in the relationships between risk factors and loss of kidney function: a general population cohort study. *BMC nephrology*. 2020;21:477-477. doi: 10.1186/s12882-020-02121-z
8. Pe De J, Jc V, Sj P-S, Hl H. Obesity and target organ damage: the kidney. *International Journal of Obesity*. 2002;26:S21. doi: 10.1038/sj.ijo.0802213
9. Gansevoort RTD, Correa-Rotter RP, Hemmelgarn BRP, Jafar THP, Heerspink HJLP, Mann JFP, Matsushita KMD, Wen CPP. Chronic kidney disease and cardiovascular risk: epidemiology, mechanisms, and prevention. *The Lancet (British edition)*. 2013;382:339-352. doi: 10.1016/S0140-6736(13)60595-4
10. Fox CS, Muntner P, Chen AY, Alexander KP, Roe MT, Cannon CP, Saucedo JF, Kontos MC, Wiviott SD. Use of Evidence-Based Therapies in Short-Term Outcomes of ST-Segment Elevation Myocardial Infarction and Non-ST-Segment Elevation Myocardial Infarction in Patients With Chronic Kidney Disease: A Report From the National Cardiovascular Data Acute Coronary Treatment and Intervention Outcomes Network Registry. *Circulation (New York, NY)*. 2010;121:357-365. doi: 10.1161/CIRCULATIONAHA.109.865352
11. Szummer K, Lundman P, Jacobson SH, Schön S, Lindbäck J, Stenestrand U, Wallentin L, Jernberg T. Relation between renal function, presentation, use of therapies and in-hospital complications in acute coronary syndrome: data from the SWEDEHEART register. *Journal of internal medicine*. 2010;268:40-49. doi: 10.1111/j.1365-2796.2009.02204.x
12. Eriksson M, Holmgren L, Janlert U, Jansson JH, Lundblad D, Stegmayr B, Soderberg S, Eliasson M. Large improvements in major cardiovascular risk factors in the population of northern Sweden: the MONICA study 1986-2009. *J Intern Med*. 2011;269:219-231. doi: 10.1111/j.1365-2796.2010.02312.x
13. Weldegiorgis M, Smith M, Herrington WG, Bankhead C, Woodward M. Socioeconomic disadvantage and the risk of advanced chronic kidney disease: Results from a cohort study

For peer review only - http://bmjopen.bmj.com/site/about/guidelines.xhtml

with 1.4 million participants. *Nephrology, dialysis, transplantation*. 2020;35:1562-1570. doi: 10.1093/ndt/gfz059
14. Stegmayr B, Lundberg V, Asplund K. The events registration and survey procedures in the Northern Sweden MONICA Project. *Scandinavian Journal of Public Health*. 2003;31:9-17. doi: 10.1080/14034950310001441
15. Lilja M, Eliasson M, Stegmayr B, Olsson T, Söderberg S. Trends in Obesity and Its Distribution: Data From the Northern Sweden MONICA Survey, 1986–2004. *Obesity (Silver Spring, Md)*. 2008;16:1120-1128. doi: 10.1038/oby.2008.8
16. Eriksson M, Carlberg B, Jansson J-H. Comparison of blood pressure measurements between an automated oscillometric device and a Hawksley random-zero sphygmomanometer in the northern Sweden MONICA study. *Blood pressure monitoring*. 2012;17:164-170. doi: 10.1097/MBP.0b013e328356ef58
17. Eliasson M, Janlert U, Jansson JH, Stegmayr B. Time trends in population cholesterol levels 1986–2004: influence of lipid-lowering drugs, obesity, smoking and educational level. The northern Sweden MONICA study. *Journal of internal medicine*. 2006;260:551-559. doi: 10.1111/j.1365-2796.2006.01730.x
18. Zeller T, Hughes M, Tuovinen T, Schillert A, Conrads-Frank A, Ruijter Hd, Schnabel RB, Kee F, Salomaa V, Siebert U, et al. BiomarCaRE: rationale and design of the European BiomarCaRE project including 300,000 participants from 13 European countries. *European journal of epidemiology*. 2014;29:777-790. doi: 10.1007/s10654-014-9952-x
19. Nyman U, Grubb A, Larsson A, Hansson L-O, Flodin M, Nordin G, Lindström V, Björk J. The revised Lund-Malmö GFR estimating equation outperforms MDRD and CKD-EPI across GFR, age and BMI intervals in a large Swedish population. *Clinical Chemistry and Laboratory Medicine (CCLM)*. 2014;52:815-824. doi: 10.1515/cclm-2013-0741
20. Levey AS, Stevens LA, Schmid CH, Zhang Y, Castro Iii AF, Feldman HI, Kusek JW, Eggers P, Lente FV, Greene T, et al. A new equation to estimate glomerular filtration rate. *Annals of internal medicine*. 2009;150:604-612. doi: 10.7326/0003-4819-150-9-200905050-00006
21. Grubb A, Horio M, Hansson L-O, Björk J, Nyman U, Flodin M, Larsson A, Bökenkamp A, Yasuda Y, Blufpand H, et al. Generation of a New Cystatin C–Based Estimating Equation for Glomerular Filtration Rate by Use of 7 Assays Standardized to the International Calibrator. *Clinical chemistry (Baltimore, Md)*. 2014;60:974-986. doi: 10.1373/clinchem.2013.220707
22. Du Bois D, Du Bois EF. A formula to estimate the approximate surface area if height and weight be known. *Archives of internal medicine (1960)*. 1916;17:863-871. doi: 10.1001/archinte.1916.00080130010002
23. Eliasson M, Eriksson M, Lundqvist R, Wennberg P, Söderberg S. Comparison of trends in cardiovascular risk factors between two regions with and without a community and primary care prevention programme. *European journal of preventive cardiology*. 2018;25:1765-1772. doi: 10.1177/2047487318778349
24. Eriksen BO, Ingebretsen OC. The progression of chronic kidney disease: A 10-year population-based study of the effects of gender and age. *Kidney International*. 2006;69:375. doi: 10.1038/sj.ki.5000058
25. Annual report 2022. Swedish Renal Registry. https://www.medscinet.net/snr/rapporterdocs/Svenskt%20Njurregister%20%C3%85rsrapport%202022%20webbversion.pdf. 2022. Accessed 2023-06-14.
26. Hallan SI, Øvrehus MA, Romundstad S, Rifkin D, Langhammer A, Stevens PE, Ix JH. Long-term trends in the prevalence of chronic kidney disease and the influence of cardiovascular risk factors in Norway. *Kidney international*. 2016;90:665-673. doi: 10.1016/j.kint.2016.04.012
27. Juutilainen A, Kastarinen H, Antikainen R, Peltonen M, Salomaa V, Tuomilehto J, Jousilahti P, Sundvall J, Laatikainen T, Kastarinen M. Trends in estimated kidney function: the FINRISK surveys. *European journal of epidemiology*. 2012;27:305-313. doi: 10.1007/s10654-012-9652-3

For peer review only

28. Turin TC, Coresh J, Tonelli M, Stevens PE, de Jong PE, Farmer CKT, Matsushita K, Hemmelgarn BR. Change in the estimated glomerular filtration rate over time and risk of all-cause mortality. *Kidney international*. 2013;83:684-691. doi: 10.1038/ki.2012.443
29. Torma E, Carlberg B, Eriksson M, Jansson J-H, Eliasson M. Long term trends in control of hypertension in the Northern Sweden MONICA study 1986-2009. *BMC Public Health*. 2015;15. doi: 10.1186/s12889-015-2280-6
30. Cortinovis M, Perico N, Ruggenenti P, Remuzzi A, Remuzzi G. Glomerular hyperfiltration. *Nature reviews Nephrology*. 2022;18:435-451. doi: 10.1038/s41581-022-00559-y
31. Zhang F, Liu H, Liu D, Liu Y, Li H, Tan X, Liu F, Peng Y, Zhang H. Effects of RAAS Inhibitors in Patients with Kidney Disease. *Current hypertension reports*. 2017;19:72-72. doi: 10.1007/s11906-017-0771-9
32. Törmä J, Lundqvist R, Eliasson M, Nilsson LM, Oskarsson V, Wennberg M. Comparison of dietary trends between two counties with and without a cardiovascular prevention programme: a population-based cross-sectional study in northern Sweden. *Public health nutrition*. 2022;25:1835-1843. doi: 10.1017/S1368980021003050
33. Copur S, Ucku D, Kanbay M. Increase in the global burden of chronic kidney disease: might it be attributable to air pollution? *Clinical kidney journal*. 2022;15:1800-1802. doi: 10.1093/ckj/sfac101
34. Wennberg M, Lundh T, Sommar JN, Bergdahl IA. Time trends and exposure determinants of lead and cadmium in the adult population of northern Sweden 1990–2014. *Environmental research*. 2017;159:111-117. doi: 10.1016/j.envres.2017.07.029
35. Bo N, James WL. Epidemiology of Nephropathia Epidemica in Sweden. *The Journal of infectious diseases*. 1987;155:269-276. doi: 10.1093/infdis/155.2.269
36. Latus J, Schwab M, Tacconelli E, Pieper F-M, Wegener D, Dippon J, Müller S, Zakim D, Segerer S, Kitterer D, et al. Clinical course and long-term outcome of hantavirus-associated nephropathia epidemica, Germany. *Emerging infectious diseases*. 2015;21:76-83. doi: 10.3201/eid2101.140861
37. Mäkelä S, Ala-Houhala I, Mustonen J, Koivisto A-M, Kouri T, Turjanmaa V, Vapalahti O, Vaheri A, Pasternack A. Renal function and blood pressure five years after Puumala virus-induced nephropathy. *Kidney international*. 2000;58:1711-1718. doi: 10.1046/j.1523-1755.2000.00332.x
38. Eric LK, Jacobien CV, Donna S, Hans LH, Dick De Z, Gary CC, Paul EDJ. Factors influencing serum cystatin C levels other than renal function and the impact on renal function measurement. *Kidney International*. 2004;65:1416. doi: 10.1111/j.1523-1755.2004.00517.x
39. Mickelsson M, Söderström E, Stefansson K, Andersson J, Söderberg S, Hultdin J. Smoking tobacco is associated with renal hyperfiltration. *Scandinavian journal of clinical and laboratory investigation*. 2021;81:622-628. doi: 10.1080/00365513.2021.1989713
40. Yamada Y, Noborisaka Y, Ishizaki M, Yamazaki M, Honda R, Yokoyama H, Kakuma T. Different association of cigarette smoking with GFR estimated from serum creatinine and that from serum cystatin C in the general population. *Clinical and experimental nephrology*. 2015;19:669-677. doi: 10.1007/s10157-014-1058-y
41. Funamoto M, Shimizu K, Sunagawa Y, Katanasaka Y, Miyazaki Y, Komiyama M, Yamakage H, Satoh-Asahara N, Takahashi Y, Wada H, et al. Serum Cystatin C, a Sensitive Marker of Renal Function and Cardiovascular Disease, Decreases After Smoking Cessation. *Circulation reports*. 2019;1:623-627. doi: 10.1253/circrep.CR-19-0052
42. Vart PMPH, Gansevoort RTMDP, Joosten MMP, Bültmann UP, Reijneveld SAP. Socioeconomic Disparities in Chronic Kidney Disease. *American journal of preventive medicine*. 2015;48:580-592. doi: 10.1016/j.amepre.2014.11.004
43. Park S, Lee S, Kim Y, Lee Y, Kang MW, Kim K, Kim YC, Han SS, Lee H, Lee JP, et al. Causal effects of education on chronic kidney disease: a Mendelian randomization study. *Clinical Kidney Journal*. 2021;14:1932-1938. doi: 10.1093/ckj/sfaa240

For peer review only - http://bmjopen.bmj.com/site/about/guidelines.xhtml

44. Fored CM, Ejerblad E, Fryzek JP, Lambe M, Lindblad P, Nyrén O, Elinder CG. Socio-economic status and chronic renal failure: a population-based case-control study in Sweden. *Nephrology, dialysis, transplantation*. 2003;18:82-88. doi: 10.1093/ndt/18.1.82
45. Baxmann AC, Ahmed MS, Marques NC, Menon VB, Pereira AB, Kirsztajn GM, Heilberg IP. Influence of Muscle Mass and Physical Activity on Serum and Urinary Creatinine and Serum Cystatin C. *Clinical journal of the American Society of Nephrology*. 2008;3:348-354. doi: 10.2215/CJN.02870707
46. Stevens LA, Schmid CH, Greene T, Li L, Beck GJ, Joffe MM, Froissart M, Kusek JW, Zhang Y, Coresh J, et al. Factors other than glomerular filtration rate affect serum cystatin C levels. *Kidney international*. 2009;75:652-660. doi: 10.1038/ki.2008.638
47. Epstein M. Alcohol's impact on kidney function. *Alcohol research & health*. 1997;21:84.
48. Hörl WH. Nonsteroidal anti-inflammatory drugs and the kidney. *Pharmaceuticals*. 2010;3:2291-2321. doi: 10.3390/ph3072291

For peer review only

Table 1. Clinical and biochemical characteristics at each evaluated time–point

	1986	1990	1994	1999	2004	2009	2014	Missing
N	1604	1535	1537	1432	1489	1390	1198	–
Age	45.7 (45.1–46.2)	45.0 (44.4–45.6)	45.3 (44.7–45.9)	45.5 (44.9–46.1)	45.6 (45.0–46.2)	46.1 (45.5–46.7)	46.6 (45.9–47.2)	0
Female sex (%)	49.4 (46.9–51.8)	50.8 (48.3–53.3)	51.3 (48.8–53.8)	51.5 (48.9–54.1)	51.8 (49.2–54.3)	51.7 (49.0–54.3)	53.0 (50.2–55.8)	0
BMI (kg/m²)*	25.0 (24.8–25.2)	25.1 (24.9–25.3)	25.5 (25.3–25.7)	25.9 (25.7–26.1)	26.3 (26.1–26.5)	26.3 (26.0–26.5)	26.5 (26.3–26.8)	51
Waist (cm)*	88.5 (88.0 – 89.1)	84.5 (83.9–85.1)	87.0 (86.4–87.7)	88.5 (87.8–89.1)	89.9 (89.3–90.6)	88.0 (87.3–88.7)	88.0 (87.2–88.7)	65
Hip (cm)*	98.0 (97.6 – 98.4)	97.6 (97.2–98.0)	99.4 (99.0–99.8)	102.8 (102.4–103.2)	100.5 (100.0–100.9)	100.8 (100.3–101.3)	98.10 (97.6–98.6)	123
SBP (mmHg)*	127 (126–128)	127 (126–128)	126 (125–127)	128 (127–129)	124 (123–125)	123 (123–124)	120 (119–121)	14
DBP (mmHg)*	80 (79–80)	80 (79–80)	79 (78–79)	79 (78–79)	77 (76–77)	78 (78–79)	79 (79–80)	14
Hypertension (%)†	33.5 (31.2–35.8)	32.3 (29.7–34.9)	31.1 (28.5–33.7)	33.6 (31.1–36.0)	30.1 (26.9–33.3)	34.8 (31.0–38.7)	21.3 (18.6–24.0)	13
Diabetes mellitus (%)‡	3.2 (2.3–4.0)	2.7 (1.9–3.5)	1.9 (1.2–2.6)	2.5 (1.7–3.3)	2.8 (1.9–3.6)	3.0 (2.1–3.8)	3.0 (2.0–4.0)	0
Active smoking (%)	31.7 (29.5–34.0)	31.5 (29.1–33.8)	29.7 (27.5–32.0)	22.3 (20.1–24.4)	21.0 (18.9–23.0)	17.0 (15.0–19.0)	12.9 (11.0–14.8)	40
Former smoking (%)	29.3 (27.1–31.5)	27.8 (25.5–30.0)	26.5 (24.3–28.7)	28.3 (26.0–30.6)	28.7 (26.4–31.1)	28.7 (26.3–31.1)	29.5 (26.9–32.1)	40
Cholesterol (mmol/L)*	6.2 (6.2–6.3)	6.1 (6.0–6.2)	5.9 (5.9–6.0)	5.6 (5.5–5.6)	5.5 (5.5–5.6)	5.4 (5.4–5.5)	5.3 (5.2–5.3)	49
hsCRP (mg/L)*	0.85 (0.80–0.90)	0.92 (0.87–0.98)	1.12 (1.05–1.18)	1.15 (1.08–1.23)	0.85 (0.80–0.90)	0.98 (0.92–1.04)	1.10 (1.03–1.17)	377
NTproBNP (ng/L)*	38.1 (36.3–40.0)	38.0 (36.2–40.0)	43.3 (41.1–45.7)	41.9 (39.8–44.2)	31.5 (29.9–33.2)	38.9 (37.0–41.1)	42.9 (40.9–45.0)	1432
Tertiary education (%)	10.8 (9.3–12.3)	16.4 (14.5–18.2)	20.7 (18.7–22.7)	24.2 (21.9–26.4)	29.1 (26.8–31.4)	32.2 (29.7–34.6)	34.5 (31.8–37.2)	–[1]
Secondary school (%)	22.2 (20.2–24.2)	22.5 (20.4–24.6)	29.3 (27.1–31.6)	35.2 (32.7–37.7)	39.4 (36.9–41.9)	41.0 (38.4–43.6)	48.5 (45.7–51.3)	–[1]
Primary school (%)	62.4 (60.0–64.8)	60.5 (58.1–63.0)	49.5 (47.0–52.0)	40.6 (38.1–43.2)	30.6 (28.3–33.0)	25.3 (23.0–27.5)	15.6 (13.6–17.7)	–[1]

For peer review only

Mean (geometric*) values with 95% confidence intervals are shown. [1] Missing data for education level = 143. †Systolic blood pressure ≥140 mmHg and/or diastolic blood pressure ≥90 mmHg, and/or antihypertensive medication. ‡ Self–reported and/or on glucose lowering medication. Primary school= 1–6 (9) years, and secondary school=7 (10)–12 years. BMI=body mass index, SBP=systolic blood pressure, DBP=diastolic blood pressure, hsCRP= high sensitivity C–reactive protein, NTproBNP=N–terminal fragment of the prohormone brain natriuretic peptide.

1

For peer review only

Table 2. Estimated glomerular filtration rate (eGFR) at each time–point

	1986	1990	1994	1999	2004	2009	2014	Missing
$eGFR_{crea}$ (LM)								
All	96.3 (95.7–96.9)	94.9 (94.2–95.6)	88.3 (87.5–89.1)	87.6 (86.7–88.5)	101.5 (100.7–102.4)	92.7 (91.7–93.6)	81.6 (80.8–82.5)	370
eGFR <60 (%)	0.7	0.6	2.1	3.5	0.9	3.1	5.2	370
Men	96.2 (95.3–97.1)	95.8 (94.9–96.8)	89.5 (88.5–90.5)	87.7 (86.5–89.0)	101.6 (100.2–102.9)	93.4 (92.1–94.8)	82.3 (81.2–83.4)	173
Women	96.4 (95.6–97.3)	94.0 (93.0–95.1)	87.1 (86.0–88.3)	87.4 (86.2–88.7)	101.5 (100.4–102.6)	91.9 (90.7–93.2)	81.1 (79.8–82.3)	197
Norrbotten	94.9 (94–95.8)	97.6 (96.6–98.5)	85.5 (84.4–86.6)	86.9 (85.9–87.9)	103.8 (102.5–105.1)	92.0 (90.6–93.5)	80.3 (79.1–81.5)	180
Västerbotten	97.8 (97–98.7)	92.5 (91.5–93.5)	91.4 (90.4–92.4)	88.3 (86.9–89.8)	99.3 (98.2–100.6)	93.4 (92.2–94.6)	83.1 (81.9–84.3)	166
$eGFR_{cysC}$ (CAPA)								
All	100.4 (99.1–101.7)	92.0 (90.9–93.1)	85.8 (84.5–87.1)	88.7 (87.4–90.0)	121.3 (119.3–123.3)	97.2 (95.7–98.7)	84.4 (83.1–85.6)	380
eGFR <60 (%)	2.4	3.3	7.4	7.5	1.5	4.0	7.7	380
Men	94.4 (92.8–96.0)	88.3 (87.0–89.6)	83.2 (81.5–85.0)	85.0 (83.3–86.7)	115.0 (112.3–117.7)	93.4 (91.5–95.4)	80.4 (78.9–81.8)	176
Women	107.0 (105.1–108.9)	95.6 (93.9–97.4)	88.3 (86.4–90.3)	92.3 (90.4–94.3)	127.4 (124.4–130.4)	100.9 (98.8–103.1)	88.1 (86.2–90.1)	204
Norrbotten	98.4 (96.7–100.2)	94.6 (93.2–96.1)	80.6 (79.3–82.0)	88.8 (87.3–90.4)	124.0 (121.1–127)	96.9 (94.7–99.2)	84.0 (82.3–85.7)	182
Västerbotten	102.5 (100.7–104.4)	89.5 (87.9–91.2)	91.7 (89.4–94.0)	88.4 (86.3–90.6)	118.5 (115.7–121.4)	97.5 (95.7–99.5)	84.8 (83.0–86.6)	174

Data shown are geometric means with 95% confidence intervals for eGFRcrea and eGFRcysC, stratified for year, sex and county (Norrbotten and Västerbotten).

For peer review only

eGFR=estimated glomerular filtration rate (ml/min/1.73 m2), crea=Creatinine, cysC=Cystatin C, LM=Lund–Malmö revised, CAPA= Caucasian, Asian, Paediatric and Adult cohort.

For peer review only

Table 3. Multivariable linear regression analysis between two different estimated glomerular filtration rates (eGFR) and clinical and biochemical variables including survey year and county

	$eGFR_{crea}$		$eGFR_{cysC}$	
	**Unstandardized β**	**Standardized β**	**Unstandardized β**	**Standardized β**
Survey	–0.004***	–0.187***	–0.001***	–0.037***
BMI (kg/m²)	0.019	0.017	–0.141***	–0.075***
Hypertension (Y/N)†	–0.013***	–0.04***	–0.017**	–0.03**
Diabetes mellitus (Y/N)‡	–0.051***	–0.046***	–0.022	–0.012
Active smoker (vs. never smoker)	0.026***	0.061***	–0.020**	–0.029**
Former smoker (vs. never smoker)	–0.004	–0.010	0.001	0.002
Cholesterol (mmol/L)	–0.118***	–0.142***	–0.128***	–0.091***
hsCRP (mg/L)	–0.023***	–0.15***	–0.064***	–0.246***
NTproBNP (ng/L)	–0.042***	–0.22***	–0.056***	–0.174***
Tertiary education (vs. primary school)	0.009	0.022	0.046***	0.065***
Secondary school (vs. primary school)	0.036***	0.094***	0.056***	0.088***
County (1=Västerbotten, 2=Norrbotten)	–0.005	–0.014	0.004	0.006

Unstandardized and standardized β-values are shown, and eGFRcrea and eGFRcysC, respectively, are dependent variables in separate multivariable analysis. eGFRcrea and eGFRcysC according to the LM and CAPA formulas respectively and were Ln–transformed. * P <0.05, ** P < 0.01, *** P < 0.001. †Systolic blood pressure ≥140 mmHg and/or diastolic blood pressure ≥90 mmHg, and/or antihypertensive medication. ‡ Self–reported and/or use of glucose lowering medication. BMI=body mass index, SBP=systolic blood pressure, DBP=diastolic blood pressure, hsCRP=high sensitivity C–reactive protein, and NTproBNP=N–terminal fragment of the prohormone brain natriuretic peptide.

For peer review only

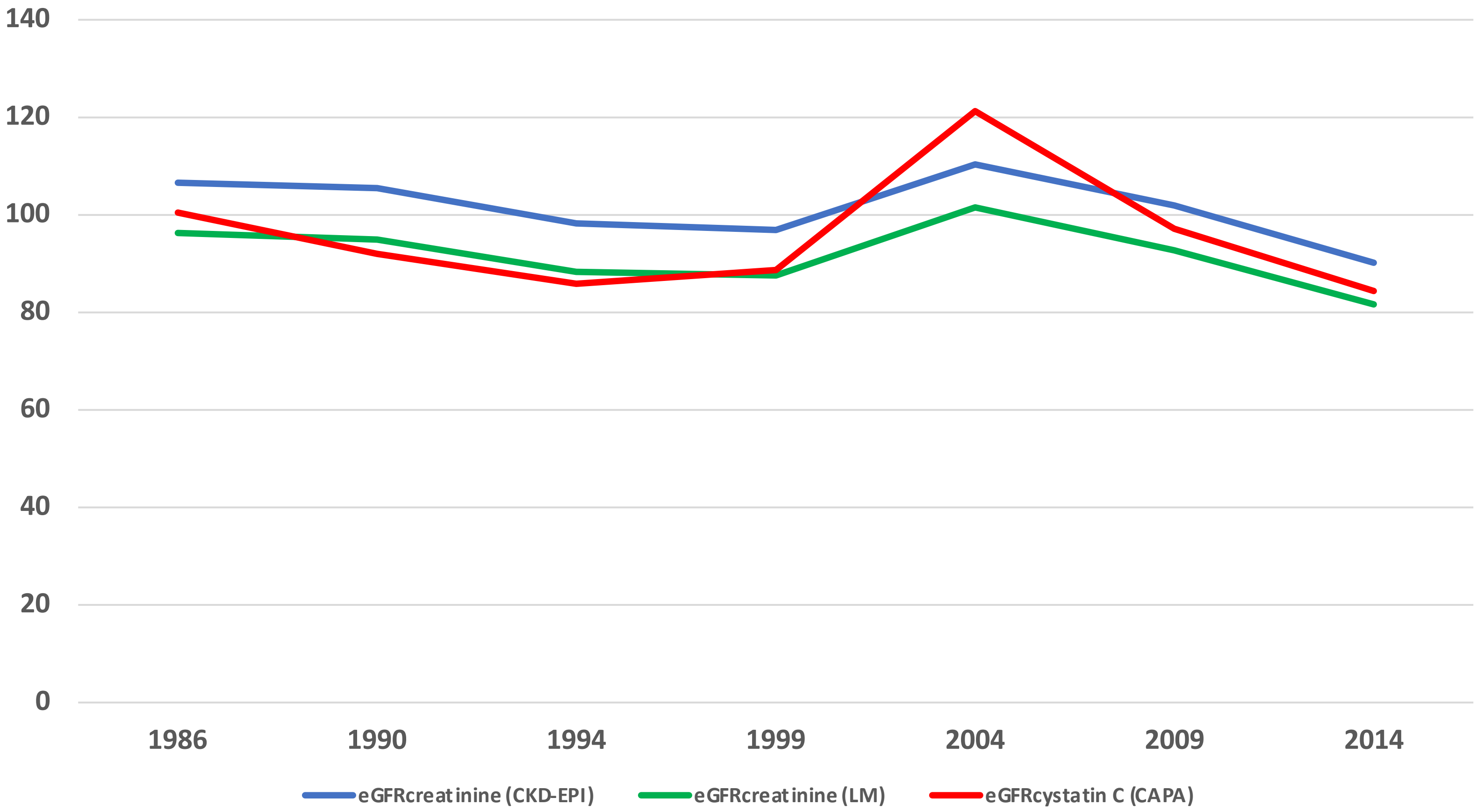
140
120
100
80
60
40
20
0
1986
1990
1994
1999
2004
2009
2014
eGFRcreatinine (CKD-EPI)
eGFRcreatinine (LM)
eGFRcystatin C (CAPA)

Trends in renal function in Northern Sweden 1986 – 2014: data from the seven cross-sectional surveys within the Northern Sweden MONICA study

Authors

Julia de Man Lapidoth[1], Johan Hultdin[2], P. Andreas Jonsson[1], Maria K Svensson[3], Maria Wennberg[4], Tanja Zeller[5,6], and Stefan Söderberg[1].

Affiliations

[1]Department of Public Health and Clinical Medicine, Medicine, Umeå University

[2]Department of Medical Biosciences, Clinical Chemistry, Umeå University

[3]Department of Medical Sciences, Renal Medicine, Uppsala University

[4]Department of Public Health and Clinical Medicine, Sustainable Health, Umeå University

[5]University Center of Cardiovascular Science, University Heart and Vascular Center Hamburg

[6]German Center of Cardiovascular Research (DZHK), Partner Seite Hamburg, Kiel, Lübeck, Hamburg/Germany

Supplementary tables 1-5

Supplementary table 1. Bivariate correlation analysis (Pearson)

Supplementary table 2. Bivariate correlation analysis (Pearson), survey 2004 excluded

Supplementary table 3. Univariable linear regression analysis

Supplementary table 4. Univariable linear regression analysis, survey 2004 excluded

Supplementary table 5. Multivariable linear regression analysis, survey 2004 excluded

For peer review only

Supplementary table 1. Bivariate correlation analysis (Pearson) between estimated glomerular filtration rate (eGFR) and clinical and biochemical variables including survey year

	$eGFR_{crea}$	$eGFR_{cysC}$
Survey year (1986–2014)	–0.142***	0
Age	–0.365***	–0.314***
BMI	–0.113***	–0.206***
Waist	–0.082***	–0.243***
Hip	–0.092***	–0.171***
SBP	–0.148***	–0.178***
DBP	–0.131***	–0.171***
Cholesterol	–0.126***	–0.146***
hsCRP	–0.206***	–0.324***
NTproBNP	–0.262***	–0.224***

$eGFR_{crea}$ and $eGFR_{cysC}$ according to the LM and CAPA formulas respectively and were Ln transformed. *** P <0.001. BMI=body mass index, SBP=systolic blood pressure, DBP=diastolic blood pressure, hsCRP= high sensitivity C–reactive protein, and NTproBNP=N–terminal fragment of the prohormone brain natriuretic peptide.

For peer review only - http://bmjopen.bmj.com/site/about/guidelines.xhtml

Supplementary table 2. Bivariate correlation analysis (Pearson), survey 2004 excluded

	$eGFR_{crea}$	$eGFR_{cysC}$
Survey year (1986–2014)	–0.222***	–0.094***
Age	–0.379***	–0.352***
BMI	–0.147***	–0.257***
Waist	–0.112***	–0.297***
Hip	–0.118***	–0.208***
SBP	–0.141***	–0.182***
DBP	–0.121***	–0.167***
Cholesterol	–0.115***	–0.139***
hsCRP	–0.199***	–0.327***
NTproBNP	–0.249***	–0.206***

$eGFR_{crea}$ and $eGFR_{cysC}$ according to the LM and CAPA formulas respectively and were Ln–transformed.

*** P <0.001. BMI=body mass index, SBP=systolic blood pressure, DBP=diastolic blood pressure, hsCRP=high sensitivity C–reactive protein, and NTproBNP=N–terminal fragment of the prohormone brain natriuretic peptide.

For peer review only

Supplementary table 3. Univariable linear regression analysis between two different estimated glomerular filtration rates (eGFR) and clinical and biochemical variables including survey year and county

	$eGFR_{crea}$		$eGFR_{cysC}$	
	**Unstandardized β**	**Standardized β**	**Unstandardized β**	**Standardized β**
Survey year (1986–2014)	–0.003***	–0.142***	0.000004	0
BMI (kg/m²)	–0.127***	–0.113***	–0.39***	–0.206***
Waist (cm)	–0.104***	–0.082***	–0.515***	–0.243***
Hip (cm)	–0.206***	–0.092***	–0.642***	–0.171***
SBP (mmHg)	–0.19***	–0.148***	–0.384***	–0.178***
DBP (mmHg)	–0.168***	–0.131***	–0.367***	–0.171***
Hypertension (Y/N)†	–0.04***	–0.125***	–0.076***	–0.141***
Diabetes mellitus (Y/N)‡	–0.088***	–0.08***	–0.112***	–0.061***
Active smoker (vs. never smoker)	0.024***	0.058***	–0.043***	–0.061***
Former smoker (vs. never smoker)	–0.013**	–0.033**	–0.023**	–0.035**
Cholesterol (mmol/L)	–0.105***	–0.126***	–0.204***	–0.146***
hsCRP (mg/L)	–0.032***	–0.206***	–0.085***	–0.324***
NTproBNP (ng/L)	–0.051***	–0.262***	–0.073***	–0.224***
Tertiary education (vs. primary school)	0.013**	0.030**	0.092***	0.130***
Secondary school (vs. primary school)	0.046***	0.120***	0.101***	0.159***
County (1=Västerbotten, 2=Norrbotten)	–0.012***	–0.033***	–0.016**	–0.027**

Unstandardized and standardized β–values are shown and $eGFR_{crea}$ and $eGFR_{cysC}$, respectively, are dependent variables in separate univariable analyses. $eGFR_{crea}$ and $eGFR_{cysC}$ according to the LM and CAPA formulas respectively and were Ln–transformed. * P <0.05, ** P < 0.01, *** P < 0.001. †Systolic blood pressure ≥140 mmHg and/or diastolic blood pressure ≥90 mmHg, and/or antihypertensive medication. ‡ Self–reported and/or use of glucose lowering medication. BMI=body mass index, SBP=systolic blood pressure, DBP=diastolic blood pressure, hsCRP=high sensitivity C–reactive protein, and NTproBNP=N–terminal fragment of the prohormone brain natriuretic peptide.

For peer review only - http://bmjopen.bmj.com/site/about/guidelines.xhtml

Supplementary table 4. Univariable linear regression analysis, survey 2004 excluded

	$eGFR_{crea}$		$eGFR_{cysC}$	
	**Unstandardized β**	**Standardized β**	**Unstandardized β**	**Standardized β**
Survey year (1986–2014)	−0.004***	−0.222***	−0.003***	−0.094***
Age (years)	−0.006***	−0.379***	−0.008***	−0.352***
Sex (1=male, 2=female)	−0.014***	−0.041***	0.084***	0.153***
BMI (kg/m²)	−0.164***	−0.147***	−0.45***	−0.257***
Waist (cm)	−0.139***	−0.112***	−0.579***	−0.297***
Hip (cm)	−0.259***	−0.118***	−0.715***	−0.208***
SBP (mmHg)	−0.178***	−0.141***	−0.36***	−0.182***
DBP (mmHg)	−0.153***	−0.121***	−0.331***	−0.167***
Hypertension (Y/N)†	−0.043***	−0.131***	−0.079***	−0.155***
Diabetes mellitus (Y/N)‡	−0.103***	−0.095***	−0.123***	−0.072***
Active smoker (vs. never smoker)	0.031***	0.075***	−0.036***	−0.057***
Former smoker (vs. never smoker)	−0.010*	−0.027*	−0.020**	−0.033**
Cholesterol (mmol/L)	−0.094***	−0.115***	−0.178***	−0.139***
hsCRP (mg/L)	−0.03***	−0.199***	−0.078***	−0.327***
NTproBNP (ng/L)	−0.047***	−0.249***	−0.061***	−0.206***
Tertiary education (vs. primary school)	0.000	0.001	0.067***	0.102***
Secondary school (vs. primary school)	0.033***	0.089***	0.082***	0.140***
County (1=Västerbotten, 2=Norrbotten)	−0.02***	−0.058***	−0.024***	−0.044***

$eGFR_{crea}$ and $eGFR_{cysC}$ according to the LM and CAPA formulas, respectively and were Ln–transformed. Unstandardized and standardized [illegible]–values are shown and $eGFR_{crea}$ and $eGFR_{cysC}$, respectively, are dependent variables in univariable analyses. * P <0.05, ** P < 0.01, *** P < 0.001. †Systolic blood pressure ≥140 mmHg and/or diastolic blood pressure ≥90 mmHg, and/or antihypertensive medication. ‡ Self–reported and/or use of glucose lowering medication. BMI=body mass index, SBP=systolic blood pressure, DBP=diastolic blood pressure, hsCRP=high sensitivity C–reactive protein, and NTproBNP=N–terminal fragment of the prohormone brain natriuretic peptide.

For peer review only

For peer review only - http://bmjopen.bmj.com/site/about/guidelines.xhtml

Supplementary table 5. Multivariable linear regression analysis, survey 2004 excluded

	$eGFR_{crea}$		$eGFR_{cysC}$	
	**Unstandardized β**	**Standardized β**	**Unstandardized β**	**Standardized β**
Survey	–0.005***	–0.26***	–0.004***	–0.128***
BMI (kg/m²)	–0.009	–0.008	–0.197***	–0.112***
Hypertension (Y/N)†	–0.015***	–0.045***	–0.021***	–0.041***
Diabetes mellitus (Y/N)‡	–0.065***	–0.06***	–0.036*	–0.021*
Active smoker (vs. never smoker)	0.025***	0.05***	–0.028***	–0.043***
Former smoker (vs. never smoker)	–0.003	–0.008	0.000	0.000
Cholesterol (mmol/L)	–0.124***	–0.152***	–0.133***	–0.103***
hsCRP (mg/L)	–0.019***	–0.127***	–0.054***	–0.227***
NT–pro–BNP (ng/L)	–0.038***	–0.201***	–0.045***	–0.152***
Tertiary education (vs. primary school)	0.006	0.015	0.041***	0.062***
Secondary school (vs. primary school)	0.032***	0.085***	0.054***	0.091***
County (1=Västerbotten, 2=Norrbotten)	–0.013***	–0.038***	–0.004	–0.007

$eGFR_{crea}$ and $eGFR_{cysC}$ according to the LM and CAPA formulas respectively and were Ln–transformed. Unstandardized and standardized [illegible]–values are shown, and $eGFR_{crea}$ and $eGFR_{cysC}$, respectively, are dependent variables in separate multivariable analysis. * P <0.05, ** P < 0.01, *** P < 0.001. †Systolic blood pressure ≥140 mmHg and/or diastolic blood pressure ≥90 mmHg, and/or antihypertensive medication. ‡ Self–reported and/or use of glucose lowering medication. BMI=body mass index, SBP=systolic blood pressure, DBP=diastolic blood pressure, hsCRP=high sensitivity C–reactive protein, and NTproBNP=N–terminal fragment of the prohormone brain natriuretic peptide.

For peer review only

STROBE 2007 (v4) Statement—Checklist of items that should be included in reports of *cross-sectional studies*

**Section/Topic**	**Item #**	**Recommendation**	**Reported on page #**
**Title and abstract**	1	(*a*) Indicate the study's design with a commonly used term in the title or the abstract	2
		(*b*) Provide in the abstract an informative and balanced summary of what was done and what was found	2
**Introduction**			
Background/rationale	2	Explain the scientific background and rationale for the investigation being reported	6-7
Objectives	3	State specific objectives, including any prespecified hypotheses	7
**Methods**			
Study design	4	Present key elements of study design early in the paper	7
Setting	5	Describe the setting, locations, and relevant dates, including periods of recruitment, exposure, follow-up, and data collection	7
Participants	6	(*a*) Give the eligibility criteria, and the sources and methods of selection of participants	7
Variables	7	Clearly define all outcomes, exposures, predictors, potential confounders, and effect modifiers. Give diagnostic criteria, if applicable	8-9
Data sources/ measurement	8*	For each variable of interest, give sources of data and details of methods of assessment (measurement). Describe comparability of assessment methods if there is more than one group	8-10
Bias	9	Describe any efforts to address potential sources of bias	12
Study size	10	Explain how the study size was arrived at	7, 11
Quantitative variables	11	Explain how quantitative variables were handled in the analyses. If applicable, describe which groupings were chosen and why	10
Statistical methods	12	(*a*) Describe all statistical methods, including those used to control for confounding	10
		(*b*) Describe any methods used to examine subgroups and interactions	12-13
		(*c*) Explain how missing data were addressed	11 and Table 1
		(*d*) If applicable, describe analytical methods taking account of sampling strategy	NA
		(*e*) Describe any sensitivity analyses	11
**Results**			

For peer review only

Participants	13*	(a) Report numbers of individuals at each stage of study—eg numbers potentially eligible, examined for eligibility, confirmed eligible, included in the study, completing follow-up, and analysed	11, table 1
		(b) Give reasons for non-participation at each stage	NA
		(c) Consider use of a flow diagram	NA
Descriptive data	14*	(a) Give characteristics of study participants (eg demographic, clinical, social) and information on exposures and potential confounders	Table 1
		(b) Indicate number of participants with missing data for each variable of interest	Table 1
Outcome data	15*	Report numbers of outcome events or summary measures	12, table 2
Main results	16	(*a*) Give unadjusted estimates and, if applicable, confounder-adjusted estimates and their precision (eg, 95% confidence interval). Make clear which confounders were adjusted for and why they were included	Table 2
		(*b*) Report category boundaries when continuous variables were categorized	Table 1 and 2
		(*c*) If relevant, consider translating estimates of relative risk into absolute risk for a meaningful time period	NA
Other analyses	17	Report other analyses done—eg analyses of subgroups and interactions, and sensitivity analyses	See above, 11
**Discussion**			
Key results	18	Summarise key results with reference to study objectives	13
Limitations	19	Discuss limitations of the study, taking into account sources of potential bias or imprecision. Discuss both direction and magnitude of any potential bias	15-16
Interpretation	20	Give a cautious overall interpretation of results considering objectives, limitations, multiplicity of analyses, results from similar studies, and other relevant evidence	15-16
Generalisability	21	Discuss the generalisability (external validity) of the study results	16-17
**Other information**			
Funding	22	Give the source of funding and the role of the funders for the present study and, if applicable, for the original study on which the present article is based	4

*Give information separately for cases and controls in case-control studies and, if applicable, for exposed and unexposed groups in cohort and cross-sectional studies.

Note: An Explanation and Elaboration article discusses each checklist item and gives methodological background and published examples of transparent reporting. The STROBE checklist is best used in conjunction with this article (freely available on the Web sites of PLoS Medicine at http://www.plosmedicine.org/, Annals of Internal Medicine at http://www.annals.org/, and Epidemiology at http://www.epidem.com/). Information on the STROBE Initiative is available at www.strobe-statement.org.

For peer review only
